# Efficient and stable noble-metal-free catalyst for acidic water oxidation

**DOI:** 10.1038/s41467-022-30064-6

**Published:** 2022-04-28

**Authors:** Sanjiang Pan, Hao Li, Dan Liu, Rui Huang, Xuelei Pan, Dan Ren, Jun Li, Mohsen Shakouri, Qixing Zhang, Manjing Wang, Changchun Wei, Liqiang Mai, Bo Zhang, Ying Zhao, Zhenbin Wang, Michael Graetzel, Xiaodan Zhang

**Affiliations:** 1 Institute of Photoelectronic Thin Film Devices and Technology, Renewable Energy Conversion and Storage Center, Solar Energy Research Center, Nankai University, Tianjin, 300350 PR China; 2 Key Laboratory of Photoelectronic Thin Film Devices and Technology of Tianjin, Tianjin, 300350 PR China; 3 Haihe Laboratory of Sustainable Chemical Transformations, Tianjin, 300192 PR China; 4 Engineering Research Center of Thin Film Photoelectronic Technology of Ministry of Education, Tianjin, 300350 PR China; 5 Collaborative Innovation Center of Chemical Science and Engineering (Tianjin), Tianjin, 300072 PR China; 6 Advanced Institute for Materials Research (WPI-AIMR), Tohoku University, Sendai, 980-8577 Japan; 7 Institute of Flexible Electronics (IFE), Northwestern Polytechnical University (NPU), Xi’an, 710072 PR China; 8 State Key Laboratory of Molecular Engineering of Polymers, Department of Macromolecular Science, Fudan University, Shanghai, 200438 PR China; 9 State Key Laboratory of Advanced Technology for Materials Synthesis and Processing, International School of Materials Science and Engineering, Wuhan University of Technology, 430070 Wuhan, PR China; 10 Laboratory of Photonics and Interfaces, Ecole Polytechnique Federale de Lausanne, Lausanne, 1015 Switzerland; 11 Canadian Light Source, Inc. (CLSI), Saskatoon, Saskatchewan Canada; 12 State Key Laboratory of Advanced Technology for Materials Synthesis and Processing, School of Materials Science and Engineering, Wuhan University of Technology, 430070 Wuhan, PR China; 13 Catalysis Theory Center, Department of Physics, Technical University of Denmark, Lyngby, 2800 Denmark

**Keywords:** Electrocatalysis, Hydrogen energy, Catalytic mechanisms

## Abstract

Developing non-noble catalysts with superior activity and durability for oxygen evolution reaction (OER) in acidic media is paramount for hydrogen production from water. Still, challenges remain due to the inadequate activity and stability of the OER catalyst. Here, we report a cost-effective and stable manganese oxybromide (Mn_7.5_O_10_Br_3_) catalyst exhibiting an excellent OER activity in acidic electrolytes, with an overpotential of as low as 295 ± 5 mV at a current density of 10 mA cm^−2^. Mn_7.5_O_10_Br_3_ maintains good stability under operating conditions for at least 500 h. In situ Raman spectroscopy, X ray absorption near edge spectroscopy, and density functional theory calculations confirm that a self-oxidized surface with enhanced electronic transmission capacity forms on Mn_7.5_O_10_Br_3_ and is responsible for both the high catalytic activity and long-term stability during catalysis. The development of Mn_7.5_O_10_Br_3_ as an OER catalyst provides crucial insights into the design of non-noble metal electrocatalysts for water oxidation.

## Abstract (web summary)

While acidic water splitting offers a renewable means to obtain renewable hydrogen fuel, the catalysts needed to oxidize water often require expensive noble metals. Here, authors show manganese oxyhalides as acidic oxygen evolution electrocatalysts.

## Introduction

Proton exchange membrane (PEM) water electrolysis has the advantages of high current density, high gas purity, and rapid system response^1–4^. The acidic water electrolyzer of PEM is a promising technique to produce hydrogen on a large-scale^5–7^. However, the catalyst for the oxygen evolution reaction (OER) is prone to corrosion in an acidic environment, leading to severe instability. Only a few noble metal-based catalysts (e.g., IrO_2_) are resistant to corrosion and are currently being used in acidic water oxidation to obtain high activity and stability^8–11^. Extensive research efforts have been devoted to improving the performance of the noble-metal catalysts or developing noble-metal free catalysts to allow their widespread deployment^12–16^.

Over the past decades, the first-row transition metal-based compounds (e.g., Mn-, Co-, Ni-based oxides) have attracted considerable interest in searching for noble-metal-free materials as high-performance acidic OER electrocatalysts^17–22^. Nevertheless, the performance of these materials remains inferior to that of the noble metal-based catalysts, and most of them exhibit relatively high overpotentials, exceeding 370 mV at a current density of 10 mA/cm^−2^ or poor durability (<50 h) (Supplementary Table 1). The existence of a stable potential window under OER potentials in acidic media (Supplementary Fig. 1) makes Mn-based materials highly attractive as a basis for a stable and active catalyst for OER^23,24^. Many Mn-based oxides have been investigated by either doping with other elements^17,25^ or optimizing morphologies^26–28^ to improve their OER activity. Unfortunately, Mn-based materials considered so far commonly suffer from rather high overpotentials and poor electronic conductivity.

In this work, we introduce Mn_7.5_O_10_Br_3_ as a low-cost and highly efficient OER catalyst in an acidic environment. The compound itself was first reported nearly 30 years ago^29–31^, but it has never been applied in electrocatalysis. We find that Mn_7.5_O_10_Br_3_ exhibits an OER overpotential (*η*) of 295 ± 5 mV at a current density of 10 mA/cm^2^ and maintains good stability under operating conditions for at least 500 h. This performance is superior to the state-of-the-art noble-metal free catalyst^23^ and comparable to that of noble-metal-containing catalysts, such as IrO_*x*_ or SrIrO_3_^32^. In-situ Raman spectroscopy combined with density functional theory (DFT) calculations were employed to investigate the catalytic activities and stabilities of different Mn-based materials (γ-MnO_2_ and Mn-O-X, X = Cl, Br). Our experiments and calculations suggest that a close-packed oxide surface forms during OER due to a self-oxidation process, resulting in excellent long-term stability and an active phase for the OER reaction. In addition, the presence of halogen ions provides the catalyst with an enhanced electron transport capacity, further enhancing the OER activity.

## Results

### Materials characterization

**Fig. 1:**
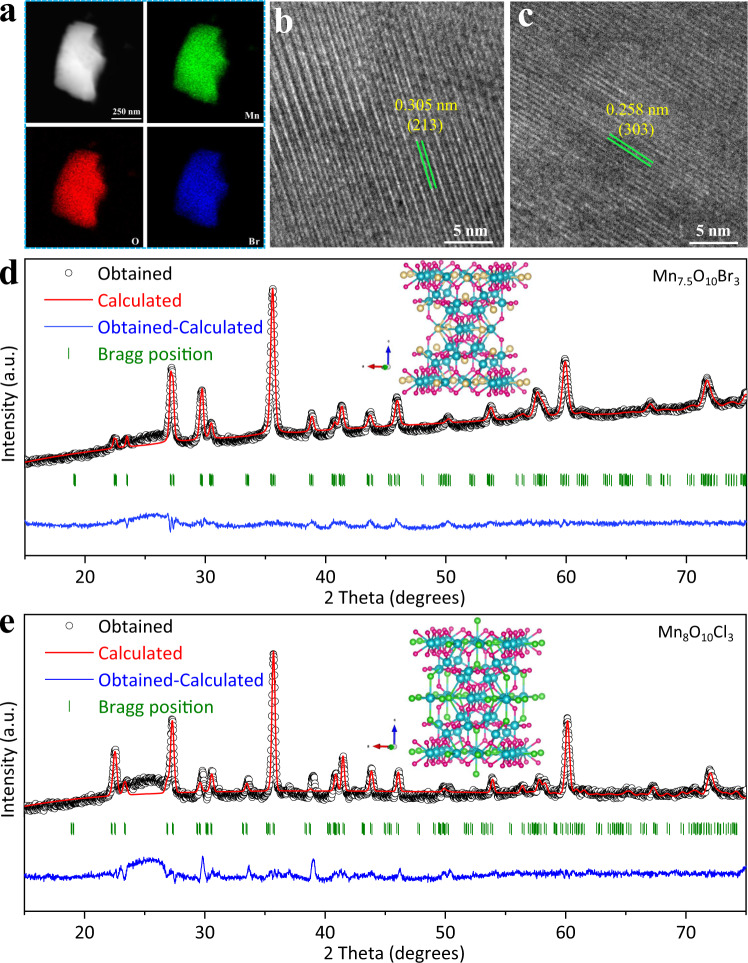
Structural characterization of Mn_7.5_O_10_Br_3_. **a** HAADF-STEM image and EDS mapping images; HRTEM image of the surface **b** (213) and **c** (303); **d** Refined X-ray diffraction (XRD) patterns of Mn_7.5_O_10_Br_3_, **e** Refined XRD patterns of Mn_8_O_10_Cl_3_.

Mn_8_O_10_Cl_3_, Mn_7.5_O_10_Br_3_, and γ-MnO_2_ were synthesized on carbon cloth through a solvothermal method. Supplementary Fig. 2 shows that the synthesized Mn_7.5_O_10_Br_3_ is uniformly coated on the carbon cloth. Energy dispersive X-ray spectroscopy (EDS) confirms (Fig. 1a) that Mn, O, and Br elements are evenly distributed in the material with negligible impurity elements, confirming its high purity. These distinctive lattice fringes are assigned to the (213) and (303) crystal planes of Mn_7.5_O_10_Br_3_ (Fig. 1b, c) with a lattice spacing of 0.305 and 0.258 nm, respectively, as determined from the high-resolution transmission electron microscopy (HRTEM). It should be mentioned that accurate determinations of catalyst surface under working conditions need operando characterizations. The phase structure of these catalysts was analyzed by X-ray diffraction (XRD) with the typical pattern presented in Fig. 1d, e. The Rietveld refinement analysis reveals that Mn_7.5_O_10_Br_3_ possesses a structure with lattice parameters of *a* = 9.3083 Å and *c* = 13.0561 Å and a space group of I4/mmm. On the other hand, the Mn_8_O_10_Cl_3_ possesses a structure with lattice parameters of *a* = 9.3757 Å and *c* = 13.0369 Å and a space group of I4/mmm. The figures of merit of these refinements are *R*_p_ = 1.421%, *R*_wp_ = 1.928% and *χ*^*2*^ = 5.677 for Mn_7.5_O_10_Br_3_ and *R*_p_ = 1.697%, *R*_wp_ = 2.327% and *χ*^*2*^ = 8.445 for Mn_8_O_10_Cl_3_. All these results indicate the successful synthesis of the target compounds.

### Evaluation of electrochemical activity

**Fig. 2:**
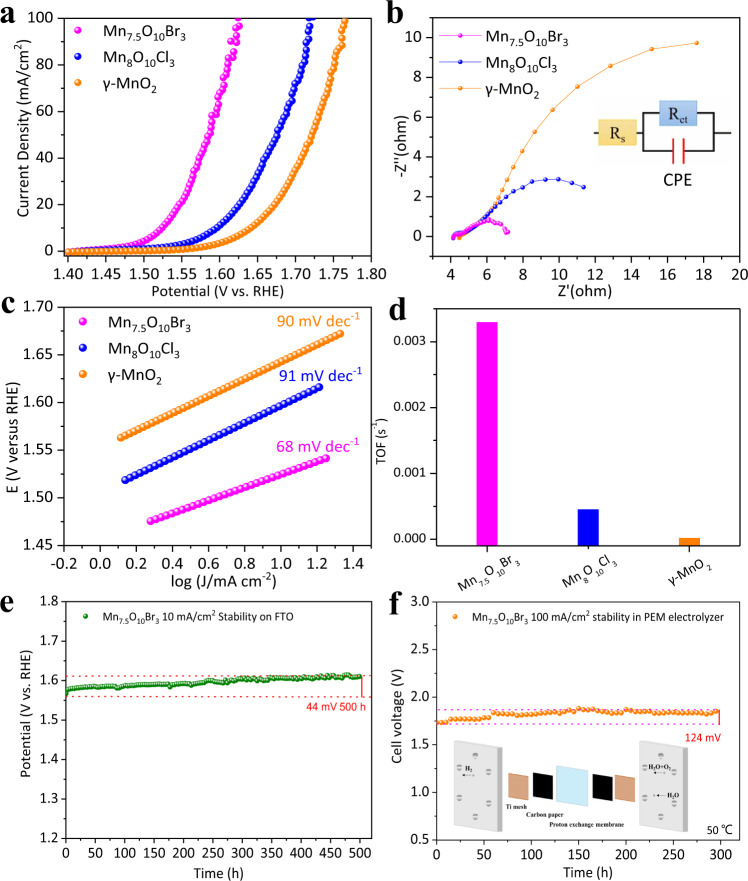
Evaluation of OER electrochemical activity. **a** LSV curves of different catalysts at 1 mV/s scan rate with iR correction. **b** Electrochemical impedance spectra (EIS) at 1.40 V (set potential). The equivalent circuit is shown (*R*_s_: series resistance; *R*_ct_: charge-transfer resistance). **c** Tafel plots of Mn_7.5_O_10_Br_3_, Mn_8_O_10_Cl_3_, and γ-MnO_2_. **d** TOF calculated from the current density at an iR-corrected overpotential of 300 mV. **e** Chronopotentiometry curves (On FTO) of Mn_7.5_O_10_Br_3_ at 10 mA cm^−2^ (25 °C). **f** Chronopotentiometry tests of the Mn_7.5_O_10_Br_3_ oxide catalyst at 100 mA cm^−2^ in the PEM electrolyzer measured at 50 °C. Inset photo: PEM electrolyzer architecture. Source data are provided as a Source Data file.

The OER activities of Mn_8_O_10_Cl_3_ and Mn_7.5_O_10_Br_3_ catalysts were evaluated using linear sweep voltammetry at a scan rate of 1 mV s^−1^ in 0.5 M sulfuric acid (pH = 0.3, Fig. 2a and Supplementary Fig. 3). The catalytic OER activity of γ-MnO_2_ was also characterized for comparison. Among the three catalysts, γ-MnO_2_ exhibits the highest overpotential of 413 ± 5 mV to reach the current density of 10 mA cm^−2^, which accords with previously reported results^23^. Mn_8_O_10_Cl_3_ shows a better activity with an overpotential of 368 ± 5 mV while Mn_7.5_O_10_Br_3_ has the lowest overpotential of 295 ± 5 mV, outperforming all previously reported noble-metal-free acidic OER electrocatalysts and even some noble metal-based electrocatalysts (Supplementary Table 1). A one-time-constant model, consisting of solution resistance in series (R_s_) with one parallel constant phase element-resistance (CPE-R_ct_) element, fits the experimental data well at all the overpotentials tested (Fig. 2b). Electrochemical impedance spectroscopy (EIS) further indicates that Mn_7.5_O_10_Br_3_ has the smallest charge transfer resistance (*R*_ct_, 7.2 Ω in Fig. 2b). Thus, it is expected to yield the fastest electrode kinetics, compared to Mn_8_O_10_Cl_3_ (12.4 Ω) and γ-MnO_2_ (26.8 Ω), which is also confirmed by their DFT + U calculated band gaps, 0.18 eV for Mn_7.5_O_10_Br_3_, 0.46 eV for Mn_8_O_10_Cl_3_, and 0.53 eV for γ-MnO_2_^33^. Due to the similar structural pattern (Supplementary Fig. 4), Mn_7.5_O_10_Br_3_ and Mn_8_O_10_Cl_3_ show somewhat comparable *R*_ct_. Tafel slope analysis (Fig. 2c) also supports the enhanced OER catalytic activity of Mn_7.5_O_10_Br_3_. We derive a value of 68 mV dec^−1^, which is much smaller than the values of 90 mV dec^−1^ and 91 mV dec^−1^ derived for γ-MnO_2_ and Mn_8_O_10_Cl_3_, respectively. The exchange current density (*I*_ex_), a key parameter to judge catalytic activity, can be estimated from the intercept of a linear Tafel plot. The *I*_ex_ values for Mn_7.5_O_10_Br_3_, Mn_8_O_10_Cl_3,_ and γ-MnO_2_ are 1.1 × 10^−4^, 7.7 × 10^−5^, and 1.5 × 10^−5^ mA cm^−2^, respectively. The Mn_7.5_O_10_Br_3_ catalyst gives a larger I_ex_ than Mn_8_O_10_Cl_3_ and γ-MnO_2_, suggesting the fastest electrode kinetics and is on the same order of magnitude as commercial RuO_2_ (3.415 × 10^−4^ mA cm^−2^)^34^. Interestingly, Mn_7.5_O_10_Br_3_ is found to have a smaller double-layer capacitance of 248.2 mF cm^−2^ (*C*_dl_) compared to Mn_8_O_10_Cl_3_ (332.9 mF cm^−2^) and γ-MnO_2_ (320.6 mF cm^−2^), as shown in Supplementary Fig. 5. Though Mn_8_O_10_Cl_3_ offers the highest density of exposed active sites per geometric electrode area, its performance is relatively inferior to that of Mn_7.5_O_10_Br_3_ even though the latter exposes a lower level of active sites.

We further calculated specific mass activities of the as-prepared catalysts at a set potential of 1.65 V (vs. RHE). Mn_7.5_O_10_Br_3_ displays the highest geometric current density of 147.77 mA cm^−2^ with a mass activity of 20.57 A g^−1^, compared with Mn_8_O_10_Cl_3_ (32.76 mA cm^−2^, 4.66 A g^−1^) and γ-MnO_2_ (12.51 mA cm^−2^, 2.05 A g^−1^). These data demonstrate the enhanced catalytic activity of Mn_7.5_O_10_Br_3_, which is more than four times higher than that of Mn_8_O_10_Cl_3_ and ten times that of γ-MnO_2_. The turnover frequency (TOF) is the most suitable metric to compare the OER activity^35–38^. The TOF was calculated based on assuming a 100% dispersion of the catalysts. From our analysis, the Mn_7.5_O_10_Br_3_ catalyst exhibits the highest TOF of 3.3 × 10^−3 ^s^−1^, exceeding that of Mn_8_O_10_Cl_3_ (4.5 × 10^−4 ^s^−1^) and γ-MnO_2_ (1.4 × 10^−5 ^s^−1^), (Fig. 2d).

The stability of these three OER catalysts was investigated by chronopotentiometry at 10 mA cm^−2^. Noteworthy, Mn_7.5_O_10_Br_3_ exhibits excellent stability, showing a rate of the potential increase of only 0.088 mV/h to maintain the current during 500 h of operation on FTO (Fig. 2e). This is comparable to other non-noble metal catalysts (See Supplementary Table 1 and the discussion there). In particular, compared to the stability of IrO_*x*_/SrIrO_3_ where no potential increase was observed within 30 h^32^, the potential of our Mn_7.5_O_10_Br_3_ merely increases by 15.6 mV over the same period. Regarding the durability of Mn_8_O_10_Cl_3_, we observed a sharp voltage rise after 75 h (Supplementary Fig. 6), suggesting it to be poorer than that of Mn_7.5_O_10_Br_3_. This is likely because the operating potential of Mn_8_O_10_Cl_3_ at 10 mA cm^−2^ is relatively higher and slightly beyond the electrochemical stability window, leading to the corrosion of the catalyst. The X-ray diffractograms of Mn_7.5_O_10_Br_3_ measured before and after the stability test show more intense characteristic peaks of the catalyst Mn_7.5_O_10_Br_3_. We suspect that those above changes may be caused by the detachment of a small amount of the catalysts from the FTO after a long period of stability (Supplementary Fig. 7), and this could be the reason for the potential increase of about 44 mV with an increased rate of 0.088 mV/h. At the same time, we find that the FTO substrate could better showcase the stability of the catalyst than carbon cloth when conducting long-term stability tests under OER working conditions (Supplementary Fig. 8). Overall, the structure of Mn_7.5_O_10_Br_3_ is preserved during long-term operation. The HRTEM after stability measurement further confirms the high stability of the catalyst (Supplementary Fig. 9). Besides, the chemical composition of the catalyst and the bonding environment of the metal center was also examined by EDS mapping and X-ray photoelectron spectroscopy (XPS) (Supplementary Figs. 10, 11). High-resolution XPS spectra of Mn 2p of electrolyte before and after stability test confirm that Mn remains inside the lattice (Supplementary Fig. 12) without any obvious dissolution for 500 h, during which the catalyst attained a number of 5.3 × 10^3^ turnovers. Moreover, the Inductively Coupled Plasma Optical Emission Spectrometry (ICP-OES) results show dissolved manganese of Mn_7.5_O_10_Br_3_ (1.05 ppm) is nearly 9× lower than that of Mn_8_O_10_Cl_3_ (8.74 ppm) and 7× lower than that of γ-MnO_2_ (6.86 ppm) (Supplementary Table 2), suggesting better stability of Mn_7.5_O_10_Br_3_. Meanwhile, no Mn ions were deposited on the counter electrode as shown by Inductively Coupled Plasma Mass Spectrometry (ICP-MS). The Faradaic efficiency for O_2_ production on three catalysts was quantified by online gas chromatography (Supplementary Fig. 13). All three samples showed high values of faradaic efficiency for oxygen evolution, namely, 99.1% ± 2.2% on Mn_7.5_O_10_Br_3_, 96.2% ± 7.1% on Mn_8_O_10_Cl_3,_ and 96.3% ± 1.0% on γ-MnO_2_ (Supplementary Figs. 14–16). The slightly smaller values for Mn_8_O_10_Cl_3_ and γ-MnO_2_ compared to Mn_7.5_O_10_Br_3_ may be due to some side reactions such as corrosion. To further evaluate the performance of Mn_7.5_O_10_Br_3_ in industrial operating systems, we next used the catalyst in a PEM electrolyzer at 50 °C (Supplementary Fig. 17). Upon applying a constant current of 100 mA cm^−2^, a cell voltage increase of 124 mV was observed during 300 h of electrolysis (Fig. 2f). Compared to γ-MnO_2_, which becomes inactivated after 12 h operation at 100 mA cm^−2^ in a PEM electrolyzer at 25 °C^23^, Mn_7.5_O_10_Br_3_ exhibits considerably superior stability at 50 °C, rendering it attractive as a noble-metal-free OER catalyst with excellent activity and stability in acidic media.

### XPS, X-ray absorption near-edge spectroscopy, and in-situ Raman analysis for mechanistic investigations

**Fig. 3:**
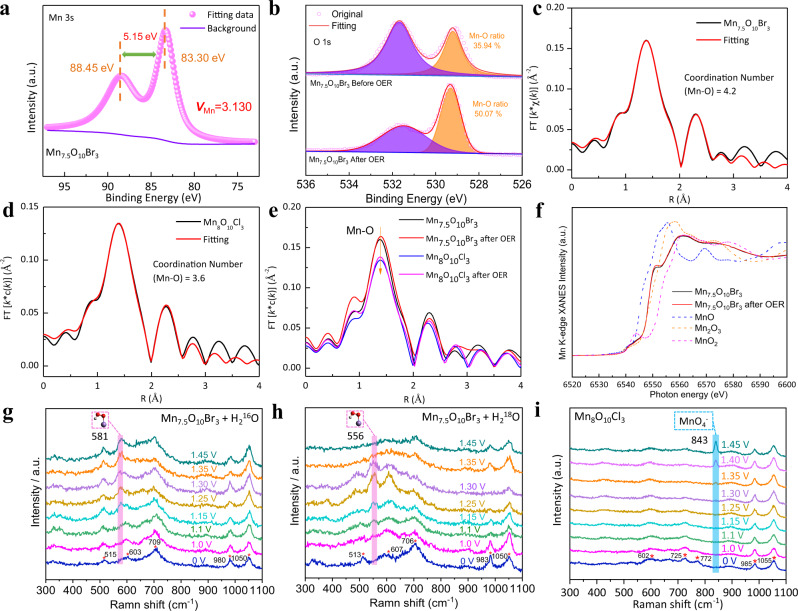
Electronic structure characterization and mechanistic investigations. High-resolution XPS spectra of Mn_7.5_O_10_Br_3_ **a** Mn *3s* and **b** O *1s*; EXAFS spectra of the Mn_7.5_O_10_Br_3_ (**c**), Mn_8_O_10_Cl_3_ (**d**) and compared with Mn_7.5_O_10_Br_3_ and Mn_8_O_10_Cl_3_ after OER stability (**e**); Normalized Mn K-edge XANES spectra of Mn_7.5_O_10_Br_3_, Mn_7.5_O_10_Br_3_ after the stability and reference materials (**f**); In-situ Raman spectra of Mn_7.5_O_10_Br_3_ catalyst on a carbon cloth in 0.5 M H_2_SO_4_ + H_2_^16^O (**g**) and 0.5 M H_2_SO_4_ + H_2_^18^O (**h**) electrolyte under different external applied potential (0–1.45 V); In-situ Raman spectra of Mn_8_O_10_Cl_3_ catalyst on a carbon cloth in 0.5 M H_2_SO_4_ + H_2_^16^O (**i**) electrolyte under different external applied potential (0–1.45 V).

Mn *3s* exchange splitting **Δ**E_3s_ (Eq. 1) is well established to be linearly correlated with the average valence state of manganese (**V**_Mn_)^39,40^, and thus can be used to determine the Mn valence state. **V**_Mn_ is determined to be 3.130 for Mn_7.5_O_10_Br_3_ and 2.939 for Mn_8_O_10_Cl_3_ based on the corresponding **Δ**E_3s_ values of 5.15 eV and 5.30 eV (Fig. 3a and Supplementary Fig. 18). **V**_Mn_ (3.137) in Mn_7.5_O_10_Br_3_ changes negligibly after longtime operations, suggesting excellent catalyst stability (Supplementary Fig. 19). Moreover, deconvolution of the high-resolution XPS spectra of O *1s* (Fig. 3b) reveals that the integral area of Mn-O area increased from 35.94% to 50.07%, compared with smaller augmentation for Mn_8_O_10_Cl_3_ (46.49–53.83%) and γ-MnO_2_ (46.77–47.97%). Thus, Mn_7.5_O_10_Br_3_ experiences the largest electro-oxidation under OER conditions (Supplementary Figs. 20, 21, and Table 3). Our DFT-calculated surface Pourbaix diagrams, discussed below, will further interpret this electro-oxidation process. To precisely analyze the valance state and the formation of close-packed oxide surface, X-ray absorption near-edge spectroscopy (XANES) and extended X-ray absorption fine structure (EXAFS) with Fourier transform was applied to characterize Mn_7.5_O_10_Br_3_ and Mn_8_O_10_Cl_3_. MnO, Mn_2_O_3,_ and MnO_2_ were employed as reference materials. The Mn K-edge absorption onsets of Mn_8_O_10_Cl_3_ and Mn_7.5_O_10_Br_3_ are close to that of Mn_2_O_3_ (Mn^3+^). Mn_7.5_O_10_Br_3_ shows a more positive absorption onset than Mn_8_O_10_Cl_3_, indicating that the Mn species in Mn_7.5_O_10_Br_3_ have a higher valence state than those in Mn_8_O_10_Cl_3_, consistent with XPS results (Supplementary Fig. 22). The increase of Mn valence state from Mn_8_O_10_Cl_3_ to Mn_7.5_O_10_Br_3_ aligns well with the increase of Mn-O coordination from 3.6 in Mn_8_O_10_Cl_3_ to 4.2 in Mn_7.5_O_10_Br_3_, as revealed by EXAFS fittings (Fig. 3c, d). The slight increase of Mn-O coordination in Mn_8_O_10_Cl_3_ and Mn_7.5_O_10_Br_3_ after OER further suggests the formation of the close-packed oxide surface of these materials (Fig. 3e). The Mn valence state shows almost no change before and after OER, indicating excellent stability of these materials (Fig. 3f and Supplementary Fig. 23). Collectively, these results give clear evidence of the formation of a close-packed oxide surface and excellent structural stability of Mn_7.5_O_10_Br_3_ in acid media.

To gain deeper insight into the OER reaction mechanism, in-situ Raman spectroscopy was utilized to better understand the active interfacial phase of the catalyst during the electrochemical process. Under acidic conditions, the OER process generally proceeds via a four-electron transfer process, i.e., water is successively oxidized to form Mn-OH, Mn-O, and Mn-OOH species, and finally, produce oxygen. Figure 3g–i, and Supplementary Fig. 24 show a series of Raman spectra for the three catalysts immersed in 0.5 M H_2_SO_4_ at selected applied potentials vs. the Ag/AgCl electrode (saturated KCl). We assign the peaks at 515, 603 and 709 cm^−1^ to Mn-O stretch vibrations^41^ of Mn_7.5_O_10_Br_3_ and peaks at 980 and 1050 cm^−1^ from SO_4_^2−^. Because of the chemical environment changes of Mn-O bond in Mn_8_O_10_Cl_3_^42,43^, we assign the peaks at 602, 725, and 772 cm^−1^ to Mn-O stretch vibrations for Mn_8_O_10_Cl_3_ and the peaks at 985 and 1055 cm^−1^ to SO_4_^2−^. Mn_7.5_O_10_Br_3_ catalyst displays a distinct Raman peak at 581 cm^−1^, whose intensity increases along with sweeping potential (Fig. 3g). The Raman peaks observed at 581 cm^−1^ can well be assigned to the Mn-OOH species^44–46^. When comparing the Raman spectra measured in H_2_^16^O and H_2_^18^O electrolyte at 1.25 V (OER onset potential), we observe a ca. 26 cm^−1^ negative shift in Mn_7.5_O_10_Br_3_ (Fig. 3h and Supplementary Fig. 25), indicating that the Mn-^18^O^18^OH adsorbed species is formed by the isotope exchange of the two ^16^O atoms (Eq. 7). According to Eq. (16), its characteristic peak will be at 556 cm^−1^. Further Raman test under constant voltage revealed that the Raman peak area around 581 cm^−1^ was directly proportional to the current density, and would become weaker with the decrease of the current density (Supplementary Fig. 26). Therefore, we can rule out the possibility of interference caused by by-products. Differently, the Raman peak at 843 cm^−1^ is assigned to the MnO_4_^−^ species^47,48^, which gradually appears on the Mn_8_O_10_Cl_3_ and γ-MnO_2_ surface under positive potential sweeping (Fig. 3i and Supplementary Fig. 24). This band intensity also increases upon augmenting the applied potential, clearly suggesting an accumulation of MnO_4_^−^ oxidative product on the Mn_8_O_10_Cl_3_ and γ-MnO_2_ surfaces. These results show that HOO* is the dominant species on the Mn_7.5_O_10_Br_3_ surface, and Mn_7.5_O_10_Br_3_ possesses better stability than Mn_8_O_10_Cl_3_ and γ-MnO_2_.

### Theoretical analysis

**Fig. 4:**
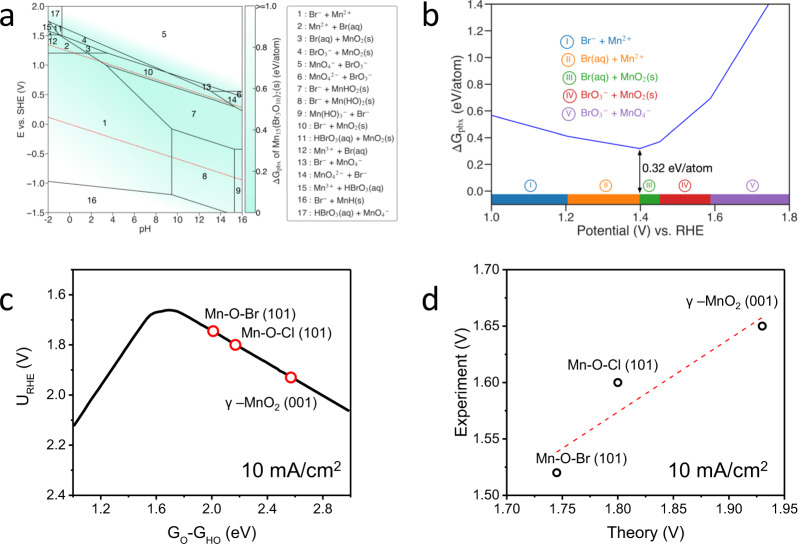
Theoretical analysis of OER stability and activity. **a** Calculated Mn-O-Br Pourbaix diagrams generated with aqueous ions concentration of 10^−4^ M at 25 °C. The Mn ions concentration used is based on the ICP-OES measurement. The Lake blue color measures the stability of Mn_7.5_O_10_Br_3_ at relevant potential and pH. The water stability window is shown in a red dashed line. **b** Calculated Pourbaix decomposition free energy (∆*G*_pbx_) of Mn_7.5_O_10_Br_3_ from the potential 1.0−1.8 V vs. RHE at pH = 0. The projection of ∆G_pbx_ onto the potential axis shows the stable species at the corresponding regions. **c** Kinetic OER activity volcano plot at 10 mA/cm^2^ as a function of G_O_-G_HO_. **d** Theory-predicted OER potentials vs. experimental values at 10 mA/cm^2^.

To further understand the stability and activity of the catalysts, DFT calculations were performed to calculate the aqueous decomposition free energy^49^ and theoretical OER limiting potentials using the recently developed microkinetic OER model^50^. The aqueous decomposition free energy (∆G_pbx_), obtained based on the DFT-calculated bulk Pourbaix diagram (Fig. 4a and Supplementary Fig. 27a), measures the catalyst stability at ambient pH and electrode potential. The calculated ∆G_pbx_ shows that Mn_7.5_O_10_Br_3_ and Mn_8_O_10_Cl_3_ can be stabilized by forming a MnO_*x*_ (e.g., MnO_2_) passivation layer on the surface at the potential of 1.40–1.60 V vs. RHE at pH = 0 (Fig. 4b and Supplementary Fig. 27b). Compared to Mn_7.5_O_10_Br_3_, the higher operating potential of Mn_8_O_10_Cl_3_ makes it suffer from a larger thermodynamic driving force to decompose, which explains our experimental observations that Mn-O-Br exhibits better stability than Mn-O-Cl at operating conditions. Figure 4c shows the derived microkinetic volcano model at 10 mA/cm^2^ as the function of G_O_-G_HO_. This microkinetic model was derived by estimating the kinetics and thermodynamics of each elementary step based on the previously discovered linear scaling relations on transition metal oxides as a function of G_HO_ (e.g., G_HO_ vs. G_O_, G_HO_ vs. G_HOO_, and G_HO_ vs. transition state energies). The potential-dependent transition state energies were calculated based on the climbing image nudged elastic band (NEB) method^51^ with explicit solvation models over different transition metal oxide surfaces using charge-extrapolation^50^. The completed modeling method, scaling relations, and details of this model are shown in Ref. ^50^. The theoretical activities of the three catalysts were analyzed by identifying the favorable surfaces, favorable surface states under operating potentials (based on surface Pourbaix diagram analysis), and active sites, with all details described in the Supplementary Discussion and Supplementary Figs. 28–31. Our surface Pourbaix diagram analyses clearly indicate that the Mn_7.5_O_10_Br_3_ and Mn_8_O_10_Cl_3_ (101) surfaces undergo a significant electro-oxidation process at the operating acidic OER conditions (Supplementary Figs. 29, 30), in excellent agreement with experiments shown in Supplementary Figs. 20, 21, and Table 3. By identifying the most favorable adsorption site (i.e., the site with the strongest HO-bonding at the surface identified by the surface Pourbaix diagram calculations in Supplementary Figs. 28–30), our kinetic volcano model allows predicting the OER potential as the function of the current density and the calculated G_O_-G_HO_ value (Fig. 4c). Our results show that the order of OER activity is Mn-O-Br > Mn-O-Cl > γ-MnO_2_. Plotting the theory-predicted OER potential vs. the experimental potential at 10 mA/cm^2^ (Fig. 4d and Supplementary Table 4), a qualitative agreement was obtained with a constant deviation of ~0.2 V on Mn-O-Br and Mn-O-Cl. This constant shifting may originate from the systemic DFT errors^52^ or the roughness of the oxide surfaces under experimental conditions. It should be noted that the formation of a more close-packed oxide surface during operating conditions will lead to an active phase of the catalyst with a weakened OER adsorbate bound with lower adsorbate-metal coordination (Supplementary Fig. 31). This suggests that the formed MnO_x_ layer not only improves the catalysts’ long-term stability but also promotes the catalytic activity, in good agreement with our experimental observations (Fig. 3).

### Outlook

We note that the Mn_7.5_O_10_Br_3_ catalyst will have a 124 mV overpotential increase after 300 h operation in the PEM electrolytic cell, which is better than most non-noble metal catalysts (Supplementary Table 1) but is less stable than iridium oxide. It should be also mentioned that the catalyst may become inactivated under certain extreme conditions, such as large current density (e.g., 1 A cm^−2^) and long-term operation (e.g., >10 k h). Researchers still need to continue the search for new materials or optimize existing structures to achieve catalytic performance comparable to or better than noble metal catalysts. Given the high cost of noble metals, especially Ir, there is also a need to evaluate the cost-effectiveness of non-precious and precious OER catalysts in the future. Notably, according to our findings, the Mn-halogen interaction and the formation of a close-packed oxide surface are key factors in improving catalytic activity and stability, which provide effective guidance for further developing non-noble metal catalysts with excellent performance.

## Discussion

In summary, we have explored the potential of manganese oxyhalides (MnXO; X = Cl, Br) as acidic oxygen evolution reaction catalysts. In particular, Mn_7.5_O_10_Br_3_ achieved superior performance in acid solution with a low overpotential of 295 ± 5 mV at a current density of 10 mA cm^−2^ and long-term stability of 500 h. XPS, XANES, and DFT results show that self-oxidation occurs on the surface of the material under OER operating conditions, consistent with the formation of a close-packed oxide surface and lower overpotentials. Our DFT calculations further show the formed close-packed oxide surface optimizes binding energies of the OER intermediate adsorbate. The Mn-halogen interaction could be the critical factor in improving the electron transport capacity. Mn_7.5_O_10_Br_3_, therefore, may serve as a viable alternative to replacing noble-metal OER catalysts. The unique structure and formation of a close-packed oxide surface, strongly related to a high OER activity and stability, would provide valuable insights for designing more effective and stable electrochemical catalysts.

## Methods

### Chemicals

Mn(NO_3_)_2_·4H_2_O (99.99%, AR, grade) was purchased from Innochem, MnBr_2_·4H_2_O was purchased from Energy Chemical (99.9% AR, grade), MnCl_2_·4H_2_O was purchased from Macklin (99.9% AR, grade), Carbon cloth substrates were purchased from CeTech (Taiwan). All chemicals were used without further purification.

### Synthesis of Mn_7.5_O_10_Br_3_

First, 143.4 mg MnBr_2_·4H_2_O was added in 300 μl H_2_O, and then treated with ultrasound until dissolved. The obtained solution was mixed in 500 μl 4 M Mn(NO3)2·4H2O solution. We transferred a volume of 300 μL of the above mixture into 1 mL centrifuge tube. Then, a 2.5 × 2.5 cm^2^ hydrophilic carbon cloth was placed on a hotplate, 300 μl of the above mixture was dropped on it, followed by calcination on a hotplate at 250 °C in the air for 5 h. After cooling to room temperature, the resultant electrode was rinsed with Milli-Q ultra-pure water and sonicated for 30 s. Finally, the electrodes were dried in an oven at 65 °C overnight before being used.

### Synthesis of Mn_8_O_10_Cl_3_ and γ-MnO_2_

First, 92.4 mg MnCl_2_·4H2O was added in 300 ul H_2_O, and then treated with ultrasonic until dissolved. The obtained solution was mixed in 500 ul 4 M Mn(NO3)2·4H2O solution. We transferred a volume of 300 μL of the above mixture into 1 mL centrifuge tube. Then, a 2.5 × 2.5 cm^2^ hydrophilic carbon cloth was placed on a hotplate, 300 ul of the above mixture was dropped on it, followed by calcination on a hotplate at 250 °C in the air for 5 h. After cooling to room temperature, the resultant electrode was rinsed with Milli-Q ultra-pure water and sonicated for 30 s. Finally, the electrodes were dried in an oven at 65 °C overnight before being used.

The γ-MnO_2_ catalyst was synthesized following a process similar to that of the Mn_8_O_10_Cl_3_ catalyst, without the addition of the MnCl_2_·4H_2_O precursors.

### Characterization

The phase composition of the samples was characterized by the XRD (PAN analytical X’Pert Pro Diffractometer with operating parameters of Cu Ka radiation (*λ* = 1.5418 Å) at 10–80°(2*θ*) at 40 kV voltage and 40 mA current) and the XPS with ESCALab220i-XL electron spectrometer. The morphology and composition of the samples were determined by FESEM under Hitachi S-4800 microscope with an acceleration voltage of 20.0 kV and TEM with a JEOLJEM-2010 instrument operating at 200 kV. Structure refinements of the XRD patterns were carried out using Rietica 1.7.7 software. In the refining process, the scale factor, background parameters, counter zero, and other general parameters are optimized. The space group of the catalyst was preliminarily determined by Le Bail thinning method, and the lattice parameters of the catalyst were approximately calculated. X-ray absorption spectra at the Mn K-edge were performed at the Soft X-ray Microcharacterization Beamline of the Canadian Light Source (CLS, Saskatoon, Saskatchewan, Canada). During measurements, X-ray beams with an incidence angle of 45° were used, and fluorescence yield was collected using silicon drift detectors. XANES and EXAFS spectra were analyzed using the Athena and Artemis softwares, respectively, included in a standardized IFEFFIT package.^53^ The manganese content of electrolyte was determined using ICP-OES (ICAP 7400) and ICP-MS (Elan drc-e). Raman spectroscopy (RTS-HiR-AM) at 532 nm excitation wavelength was used. In situ Raman (Supplementary Fig. 26) was normalized with the integral area of the characteristic peak of SO_4_^2−^ around 1052 cm^−1^, and then we performed peak fitting to divide the characteristic peak into two peaks around 581 cm^−1^ and 603 cm^−1^, and then processed the Raman peak around 581 cm^−1^ corresponding to 1.45 V. All fitting standard deviations (*χ*^2^) were controlled below 0.1. The average valence state of manganese (*V*_Mn_) was calculated to the equation^40^:

1 $${V}_{{{{{{\rm{Mn}}}}}}}=9.69-1.27\triangle {E}_{3s}/{{{{{\rm{eV}}}}}}$$

Where **V**_Mn_ is the average valence state of manganese, **Δ**E_3s_ is the measured Mn 3s level splitting.

### Electrochemical characterization

The electrochemical performance was tested on an electrochemical station (Autolab PGSTAT302N) with a built-in EIS analyzer. The electrodes were pretreated as anodes in sulfuric acid solution (0.5 M H_2_SO_4_) at a current density of 10 mA cm^−2^ for 2 h. Afterward, the working electrode was a carbon cloth (area: 0.5 cm^2^). The carbon electrode was used as the counter electrode. The Ag/AgCl (with saturated KCl was used as the filling solution) electrode served as the reference electrode. The current density (*j*) versus potential (*U*) curves were obtained with a sweep rate of 1 mV s^−1^ from 1.8 V to 1.2 V (vs. RHE) in 0.5 M H2SO4. EIS measurements were conducted in a static solution at an overpotential of 170 mV from 0.1 Hz to 100 kHz with an amplitude of 10 mV. The electrocatalyst’s C_dl_ was measured using cyclic voltammograms in a no Faradaic reaction potential window (0.99–1.09 V vs. RHE) at scanning rates of 2, 4, 6, 8, and 10 mV s^−1^. At 1.04 V vs. RHE, the curve of current density difference △*J* = (*J*_1_ − *J*_2_)/2 with scanning rate is a straight line, and the slope is C_dl_. The mass activity was calculated based on the catalyst loadings on the carbon cloth working electrode with an area of 0.5 cm^−2^ were 3.592 mg for Mn_7.5_O_10_Br_3_, 3.516 mg for Mn_8_O_10_Cl_3_, and 3.056 mg for γ-MnO_2_. All experiments were carried out at ambient temperature (25 ± 2 °C), and the electrode potential was converted to the RHE scale according to given equation:

2 $$E\left({RHE}\right)={{{{{\rm{E}}}}}}\left({Ag}/{AgCl}\right)+0.197{{{{{\rm{V}}}}}}+0.059{{{{{\rm{\times }}}}}}{{{{{\rm{PH}}}}}}$$

### IR correction

Ohmic loss correction was carried out for polarization curves on different support surfaces. The correction was done according to the given equation^54^:

3 $${E}_{{{{{{\rm{corrected}}}}}}}={{{{{\rm{E}}}}}}-{{{{{\rm{iR}}}}}}$$

Where *E*_corrected_ is the potential corrected by IR, *E* is the potential measured in experiment, and *R* is the series resistance measured.

**Acid OER pathway**

4 $$M+{{{{{\rm{H}}}}}}_{2}{{{{{\rm{O}}}}}}\to {{{{{\rm{MOH}}}}}}+{{{{{\rm{H}}}}}}^{+}+{e}^{-}$$

5 $${MOH}\to {{{{{\rm{MO}}}}}}+{H}^{+}+{e}^{-}$$

6 $${MO}+{{{{{\rm{H}}}}}}_{2}{{{{{\rm{O}}}}}}\to {{{{{\rm{MOOH}}}}}}+{H}^{+}+{e}^{-}$$

7 $${MOOH}\to {{{{{\rm{M}}}}}}+{{O}_{2}+H}^{+}+{e}^{-}$$

### TOF calculations of Mn_7.5_O_10_Br_3,_ Mn_8_O_10_Cl_3,_ and γ-MnO_2_ in this work

The TOFs of these catalysts were calculated using the following equation^55^:

8 $${{{{{\rm{TOF}}}}}}=\frac{{{{{{\rm{J}}}}}}* {{{{{\rm{A}}}}}}* {{{{{\rm{\eta }}}}}}}{4* {{{{{\rm{F}}}}}}* {{{{{\rm{n}}}}}}}$$

*J* is obtained at iR-corrected overpotential = 300 mV, normalized by the geometric area of carbon cloth (0.5 cm^2^); *R* is the series resistance obtained from EIS fitting. *A* is the geometric area of carbon cloth (0.5 cm^2^). *F* is the Faraday constant, and *η* is the Faradaic efficiency. *n* is the mole number of loading manganese atoms on the electrode.

### Faradaic efficiency of oxygen

Online gas chromatography (Trace GC Ultra, Thermo Scientific) was used to test the Faraday efficiency of oxygen. The details of the instrument have been shown in previous work^56^. The gas chromatography was calibrated by injecting a known concentration of oxygen gas dissolved in the Helium matrix (99.9999%). The peak area was plotted against the concentration (in ppm) of oxygen, resulting in a linear fitting curve, which was used for quantifying the amount of oxygen during the reaction on three samples (Supplementary Fig. 13).

During the electrolysis process, 99.9999% Helium is used as an inert gas, and the evolved oxygen gas was continuously discharged into the sample circuit at a constant rate of 20 sccm. Three samples were tested for oxygen evolution at 10 mA cm^−2^ for 100 min. The average Faraday efficiency is calculated for 7–8 injections during electrolysis (except for the first injection, which takes about 20 min to equilibrate the headspace).

The Faradaic efficiency is calculated as follows:

9 $${{{{{\rm{Faradaic}}}}}}\,{{{{{\rm{efficiency}}}}}}\;\left( \% \right)=\frac{Q}{{Q}_{{{{{{\rm{o}}}}}}}}{{\times }}100 \%$$

*Q* represents the charge for producing oxygen

*Q*_o_ represents charge used for electrolysis

Each injection represents the amount of oxygen produced during a time period of *t*_o_,

10 $${t}_{{{{{{\rm{o}}}}}}}=\frac{V}{v}$$

*V* represents the volume of a sample loop

*v* represents the flow rate

The total charge used for electrolysis (*Q*_o_) is calculated as

11 $${Q}_{{{{{{\rm{o}}}}}}}={\int }_{\!\!\!t}^{{t}+{t}_{{{{{{\rm{o}}}}}}}}{I}{{\times }}{{{{{\rm{dt}}}}}}={I}_{{{{{{\rm{o}}}}}}}{{\times }}{t}_{{{{{{\rm{o}}}}}}}={I}_{{{{{{\rm{o}}}}}}}{{\times }}\frac{V}{v}$$

*I*_o_ represents average current during injection

The number of moles oxygen (*n*_o_) for each sampling is calculated as:

12 $${n}_{{{{{{\rm{o}}}}}}}=\frac{{P}{{\times }}{V}\left( \% \right){{\times }}{V}}{{R}{{\times }}{T}}$$

*V*_o_(%) represents the volumetric concentration of the gas, which is determined by the peak area of the oxygen peak and the calibration curve

*T* represents the temperature of the test (*T* = 293 K)

*P* represents the pressure during the test (*P* = 1.01 × 10^5 ^Pa)

*R* represents the ideal gas constant (*R* = 8.3145 J mol^−1^ K^−1^)

The charge used for producing oxygen (*Q*) is calculated as:

13 $${{Q}}={{n}}_{{{{{{\rm{o}}}}}}}{{\times }}{N}{{\times }}{F}=\frac{{P}{{\times }}{V}\left( \% \right){{\times }}{V}}{{R}{{\times }}{T}}{{\times }}{N}{{\times }}{F}$$

*N* represents the number of electrons/holes passed for one oxygen molecule (*N* = 4)

*F* represents the Faraday constant (*F* = 96485.3 C mol^−1^)

Combining Eqs. (9), (11), and (13), the Faradaic efficiency is calculated as:

14 $${{{{{\rm{Faradaic}}}}}}\; {{{{{\rm{efficiency}}}}}}\left( \% \right)=\frac{\frac{{P}{{\times }}{V}\left( \% \right){{\times }}{V}}{{R}{{\times }}{T}}{{\times }}{{{{{\rm{N}}}}}}{{\times }}{F}}{{{I}}_{{{{{{\rm{o}}}}}}}{{\times }}\frac{{V}}{{v}}}{{\times }}100 \% =\frac{{P}{{\times }}{V}( \% ){{\times }}{{N}}{{{{{\rm{\times }}}}}}{F}{{{{{\rm{\times }}}}}}{v}}{{R}{{\times }}{T}{{\times }}{{I}}_{{{{{{\rm{o}}}}}}}}{{\times }}100 \%$$

### Raman shift calculation of isotope lab

The Raman shift in 0.5 M H_2_SO_4_ (H_2_^18^O) of Mn_7.5_O_10_Br_3_ catalyst was calculated using the following equation^57^:

The Raman characteristic peak after the exchange of one ^18^O atom is σ_1_

15 $${{{{{{\rm{\sigma }}}}}}}_{1}=\frac{{{{{{\rm{\vartheta }}}}}}\left({18}_{{{{{{\rm{O}}}}}}\,-\,{{{{{\rm{OH}}}}}}}\right)}{{{{{{\rm{\vartheta }}}}}}\left({16}_{{{{{{\rm{O}}}}}}\,-\,{{{{{\rm{OH}}}}}}}\right)}=\frac{\sqrt{\frac{{{{{{{{\rm{m}}}}}}}_{18}}_{{{{{{\rm{O}}}}}}}+{{{{{{{\rm{m}}}}}}}_{16}}_{{{{{{\rm{OH}}}}}}}}{{{{{{{{\rm{m}}}}}}}_{18}}_{{{{{{\rm{O}}}}}}}{{\times }}{{{{{{{\rm{m}}}}}}}_{16}}_{{{{{{\rm{OH}}}}}}}}}}{\sqrt{\frac{{{{{{{{\rm{m}}}}}}}_{18}}_{{{{{{\rm{O}}}}}}}+{{{{{{{\rm{m}}}}}}}_{16}}_{{{{{{\rm{O}}}}}}}}{{{{{{{{\rm{m}}}}}}}_{18}}_{{{{{{\rm{O}}}}}}}{{\times }}{{{{{{{\rm{m}}}}}}}_{16}}_{{{{{{\rm{O}}}}}}}}}}{{{{{\rm{\sigma }}}}}}=0.9843{{{{{\rm{\sigma }}}}}}$$

The Raman characteristic peak after the exchange of two ^18^O atom is σ_2_

16 $${{{{{{\rm{\sigma }}}}}}}_{2}=\frac{{{{{{\rm{\vartheta }}}}}}\left({18}_{{{{{{\rm{O}}}}}}}-{18}_{{{{{{\rm{OH}}}}}}}\right)}{{{{{{\rm{\vartheta }}}}}}\left({16}_{{{{{{\rm{O}}}}}}}-{16}_{{{{{{\rm{OH}}}}}}}\right)}=\frac{\sqrt{\frac{{{{{{{{\rm{m}}}}}}}_{18}}_{{{{{{\rm{O}}}}}}}+{{{{{{{\rm{m}}}}}}}_{18}}_{{{{{{\rm{OH}}}}}}}}{{{{{{{{\rm{m}}}}}}}_{18}}_{{{{{{\rm{O}}}}}}}{{\times }}{{{{{{{\rm{m}}}}}}}_{18}}_{{{{{{\rm{OH}}}}}}}}}}{\sqrt{\frac{{{{{{{{\rm{m}}}}}}}_{18}}_{{{{{{\rm{O}}}}}}}+{{{{{{{\rm{m}}}}}}}_{16}}_{{{{{{\rm{O}}}}}}}}{{{{{{{{\rm{m}}}}}}}_{18}}_{{{{{{\rm{O}}}}}}}{{\times }}{{{{{{{\rm{m}}}}}}}_{16}}_{{{{{{\rm{O}}}}}}}}}}{{{{{\rm{\sigma }}}}}}=0.9573{{{{{\rm{\sigma }}}}}}$$

### Proton exchange membrane (PEM) electrolyzer

Membrane electrode assemblies (MEAs) were prepared using a Nafion^®^117 polymer membrane (DuPont, thickness 177.8 µm, N117). Before MEAs preparation, the N117 membrane was boiled separately for half an hour in the following solutions to remove possible contaminants and ensure complete protonation: first 3 wt.% H_2_O_2_, then Milliq ultra-pure water, then 1.0 M H_2_SO_4_, and finally Milliq ultra-pure water. Finally, the N117 film is dried in a 40 °C oven for several hours before use. The MEAs were prepared using Pt as the cathode for the hydrogen evolution reaction and Mn_7.5_O_10_Br_3_ as the anode for the oxygen evolution reaction. The OER catalyst of Mn_7.5_O_10_Br_3_ was directly synthesized on carbon paper (TGP-H-060 purchased from Adrian Electronic Technology Co., LTD). The Pt/c (20 wt% Pt on carbon black purchased from innochem) deposited onto carbon paper was used as the HER catalyst. The mass loadings were controlled at 3.2 mg ± 0.5 cm^−2^ of Mn_7.5_O_10_Br_3_ and 0.2 mg cm^−2^ of Pt for anodic and cathodic catalysts, respectively. The effective area of the MEA was 2 cm × 2 cm (4 cm^2^). Electrolysis tests were conducted using a single cell PEM electrolyzer. The titanium meshes were used as gas diffusion layers for both the anode and cathode. During the test, the cell was maintained at 50 °C, and the pre-heated DI water was fed to the anode at a flow rate of 10 ml min^−1^ (pipe diameter 4.8 mm).

### DFT calculations

Spin-polarized DFT + U calculations were performed using the VASP code^58^ with the U_eff_ value of 3.9 eV for Mn 3d, which is in line with the Materials Project^33^. Electronic exchange and correlations were described by the generalized gradient approximation method with the revised Perdew–Burke–Ernzerhof functional^52^. Core electrons were described by the projector augmented wave method. Valence electrons were described by expanding the Kohn-Sham wave functions in a plane-wave basis set. The energy cutoff for all of the calculations was set as 400 eV. Convergence was defined when all the forces of each atom were below 0.05 eV per Å. (3 × 3 × 1) k-point meshes were used to sample the Brillouin zone using the Gamma point sampling. The bulk Pourbaix diagram was calculated using the Strongly Constrained and Appropriately Normed (SCAN) functional^59^, which has been recently shown to accurately predict the aqueous stability of solids^24^. The detailed construction of Pourbaix diagram was provided in Ref. 60^60^ and Ref. ^24^. In SCAN calculations, the plane wave energy cutoff was 520 eV. The electronic energy and structure relaxation were converged to 10^−5 ^eV and 0.02 eV/Å, respectively. The Brillouin zone was integrated with a k-point density of 1000 per reciprocal atom. The microkinetic OER activity model was developed based on the methods and scaling relations described in Ref. ^50^.

## Supplementary information

Supplementary Information

Description of Additional Supplementary Files

Supplementary Movie 1

## Data Availability

All data necessary to support the findings of this study are available in the Supplementary Information. The raw data are available from the corresponding author upon reasonable request. Source data are provided in this paper.

## Source data

Source Data
