# Supplementary material for: Efficient and stable noble-metal-free catalyst for acidic water oxidation
Source: Nat Commun. 2022 Apr 28;13:2294. doi: 10.1038/s41467-022-30064-6 (PMC9050677; doi:10.1038/s41467-022-30064-6)
Supplement: Supplementary file 1 — Supplementary Information [file 41467_2022_30064_MOESM1_ESM.pdf]

Supplementary Information

Efficient and Stable Noble-Metal-Free Catalyst for Acidic Water Oxidation

Sanjiang Pan [1,2,3,4,5†], Hao Li [6†], Dan Liu [7], Rui Huang [8], Xuelei Pan [9], Dan Ren [10], Jun Li [10], Mohsen Shakouri [11], Qixing Zhang [1,2,3,4,5], Manjing Wang [1,2,3,4,5], Changchun Wei [1,2,3,4,5], Liqiang Mai [12], Bo Zhang [8], Ying Zhao [1,2,3,4,5], Zhenbin Wang [13]*, Michael Graetzel [1,10]*, Xiaodan Zhang [1,2,3,4,5]*

[1] Institute of Photoelectronic Thin Film Devices and Technology, Renewable Energy Conversion and Storage Center, Solar Energy Research Center, Nankai University, Tianjin 300350, PR China

[2] Key Laboratory of Photoelectronic Thin Film Devices and Technology of Tianjin, Tianjin 300350, PR China

[3] Haihe Laboratory of Sustainable Chemical Transformations, Tianjin 300192, P. R. China

[4] Engineering Research Center of Thin Film Photoelectronic Technology of Ministry of Education, Tianjin 300350, P.R. China

[5] Collaborative Innovation Center of Chemical Science and Engineering (Tianjin), Tianjin 300072, China

[6] Advanced Institute for Materials Research (WPI-AIMR), Tohoku University, Sendai 980-8577, Japan

[7] Institute of Flexible Electronics (IFE), Northwestern Polytechnical University (NPU), Xi'an 710072, China

[8] State Key Laboratory of Molecular Engineering of Polymers, Department of Macromolecular Science, Fudan University, Shanghai 200438, China

[9] State Key Laboratory of Advanced Technology for Materials Synthesis and Processing, International School of Materials Science and Engineering, Wuhan University of Technology, 430070 Wuhan, P.R. China

[10] Laboratory of Photonics and Interfaces, Ecole Polytechnique Federale de Lausanne, Lausanne 1015, Switzerland

[11] Canadian Light Source, Inc. (CLSI), Saskatoon, Saskatchewan, Canada

[12] State Key Laboratory of Advanced Technology for Materials Synthesis and Processing, School of Materials Science and Engineering, Wuhan University of Technology, 430070 Wuhan, P. R. China

[13] Catalysis Theory Center, Department of Physics, Technical University of Denmark, Lyngby 2800, Denmark

*E-mail: zwang@fysik.dtu.dk, michael.graetzel@epfl.ch, xdzhang@nankai.edu.cn

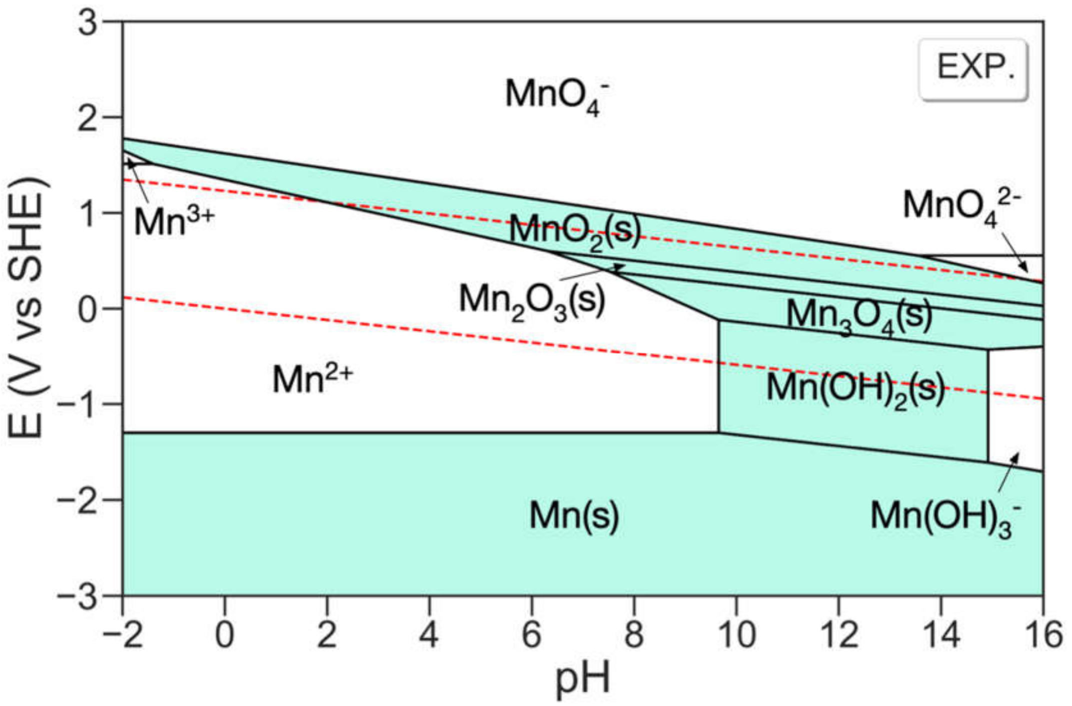

Supplementary Figure 1. The experimental Mn-O-H Pourbaix diagram generated with aqueous ion concentration of 10^{-4} M at 25 °C. The Mn ions concentration of 10^{-4} M was selected based on the ICP-OES measurement (Table S2). Regions with solid are shaded in Lake blue. The water stability window is shown in red dashed line. The experiment data were obtained from Reference 1.

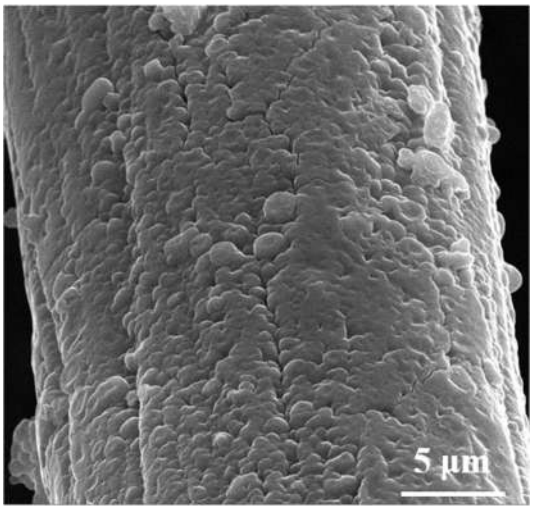

Supplementary Figure 2. Morphology of $Mn_{7.5}O_{10}Br_3$. SEM image of $Mn_{7.5}O_{10}Br_3$ coated on carbon cloth.

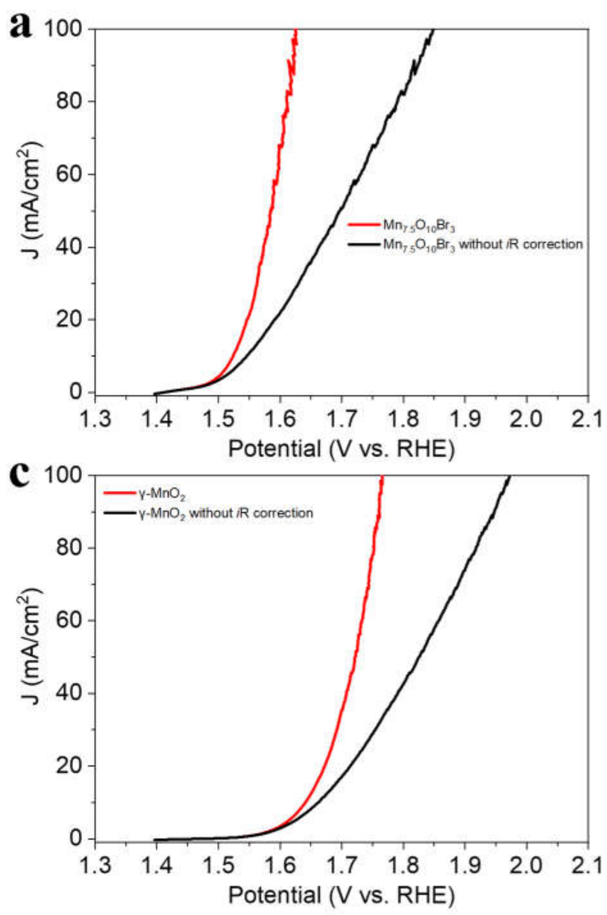

Supplementary Figure 3. Activity comparison between with iR and without iR compensation. Linear sweep voltammograms of $Mn_{7.5}O_{10}Br_3$ (a), $Mn_8O_{10}Cl_3$ (b), and γ-MnO_2 (c) loaded on carbon cloth in 0.5 M H_2SO_4 with (black line) and without iR correction (red line).

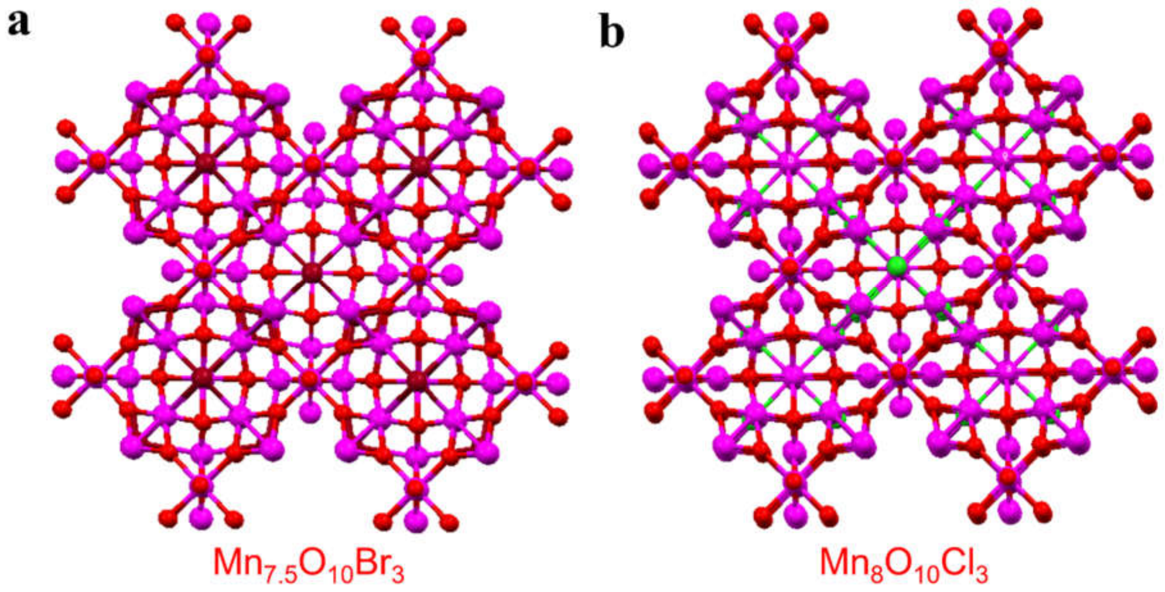

Supplementary Figure 4. Material structure. Crystal structure diagram of $Mn_{7.5}O_{10}Br_3$ (a), $Mn_8O_{10}Cl_3$ (b).

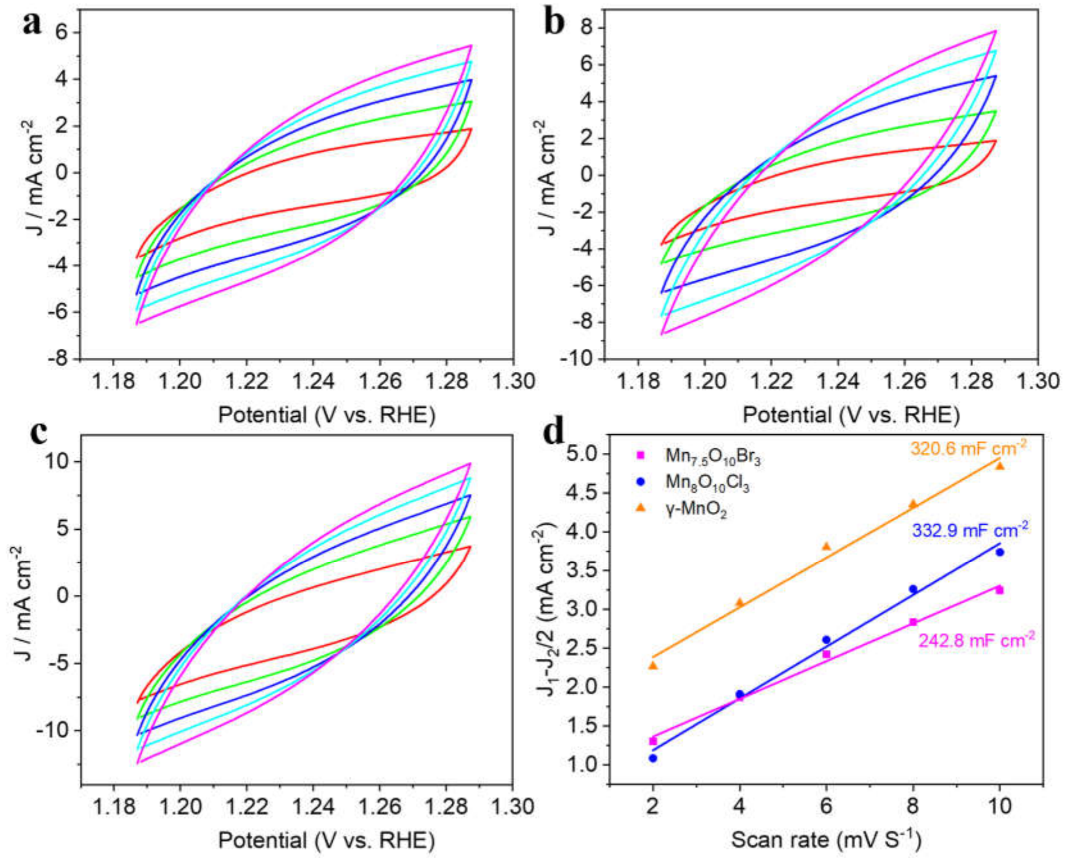

Supplementary Figure 5. Electrochemical double-layer capacitance measurements. cyclic voltammetry curves of: (a) $Mn_{7.5}O_{10}Br_3$ from 1.18 to 1.28 V vs. RHE at scanning rates of 2, 4, 6, 8, to 10 mV s^{-1}. (b) $Mn_8O_{10}Cl_3$ from 1.18 to 1.28 V vs. RHE at scanning rates of 2, 4, 6, 8, to 10 mV s^{-1} (c) γ-MnO_2 from 1.18 to 1.28 V vs. RHE at scanning rates of 2, 4, 6, 8, to 10 mV s^{-1}. (d) The fitting plots showing C_{dl} for $Mn_{7.5}O_{10}Br_3$, $Mn_8O_{10}Cl_3$ and γ-MnO_2.

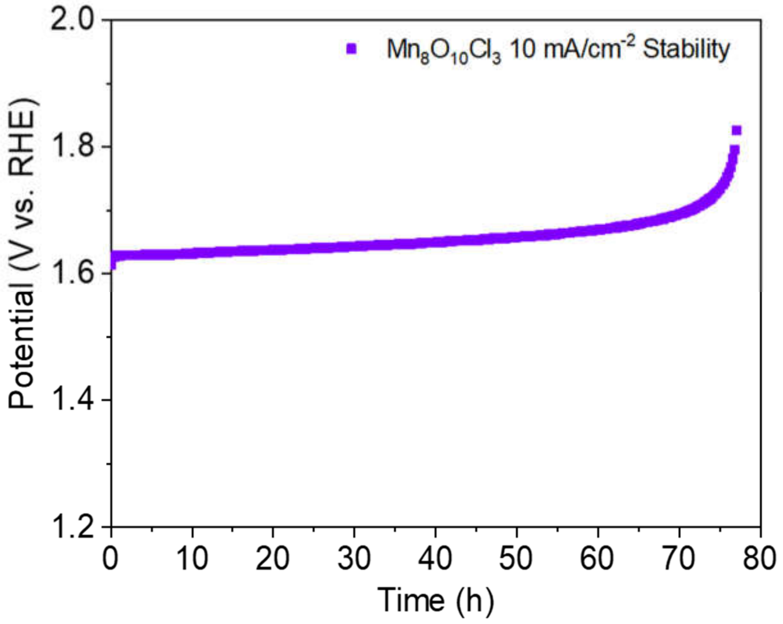

Supplementary Figure 6. OER stability test of $Mn_8O_{10}Cl_3$. Chronopotentiometry curves obtained from $Mn_8O_{10}Cl_3$ with a constant current density of 10 mA/cm^2.

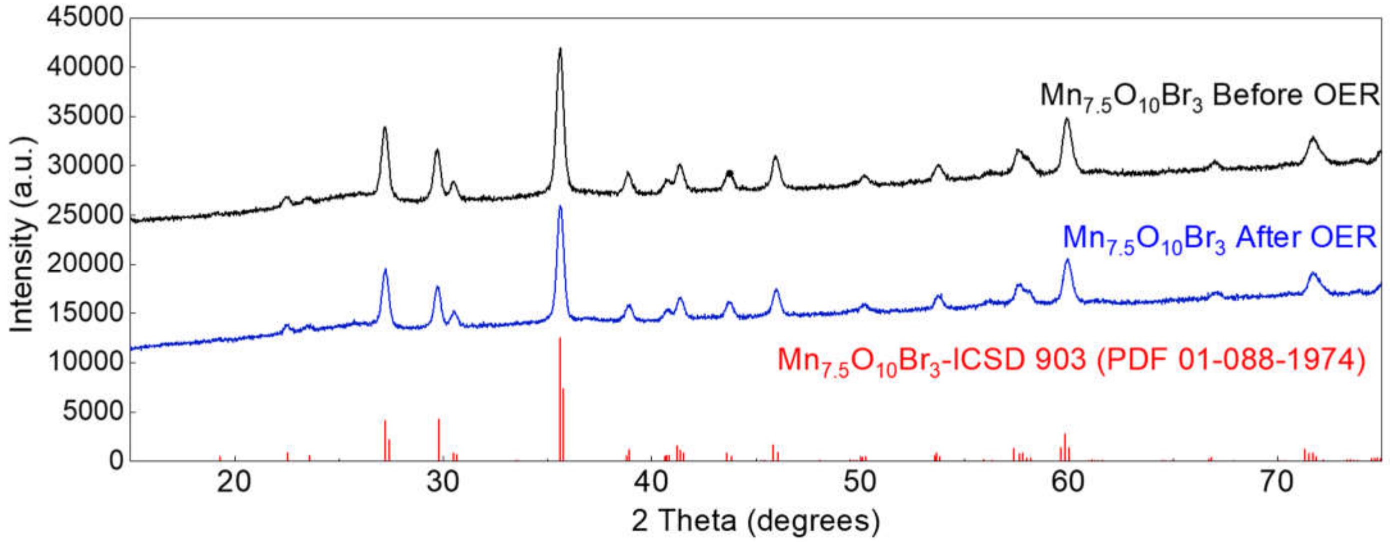

Supplementary Figure 7. Structural stability. XRD pattern of $Mn_{7.5}O_{10}Br_3$ before (black line) and after (blue line) the stability test on carbon cloth.

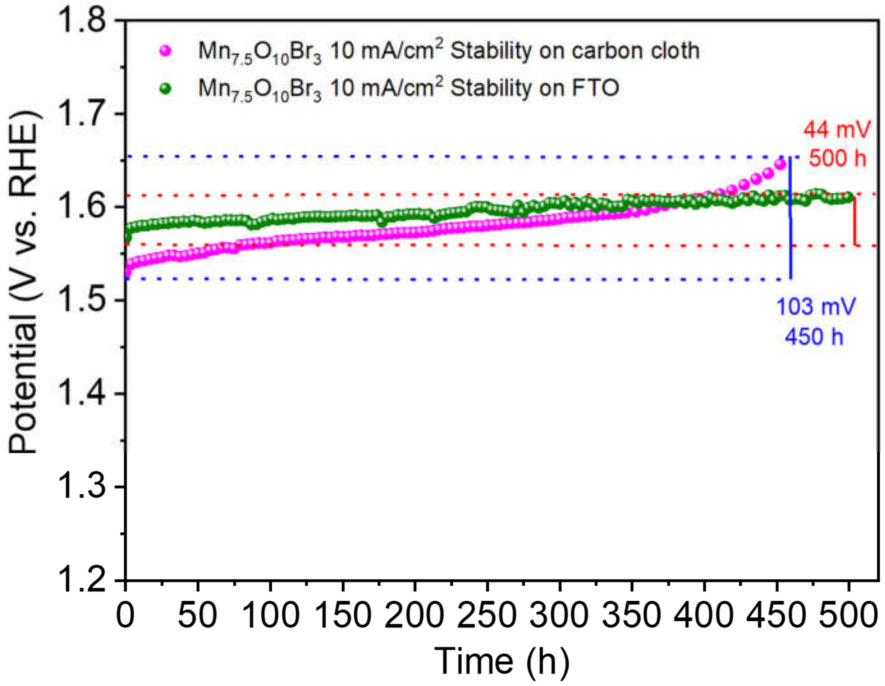

Supplementary Figure 8. Chronopotentiometry tests of the $Mn_{7.5}O_{10}Br_3$ catalyst at 10 mA cm^{-2} on carbon cloth and FTO. The relatively larger increase of potentials on carbon cloth is likely because of the substrate corrosion under OER working conditions.

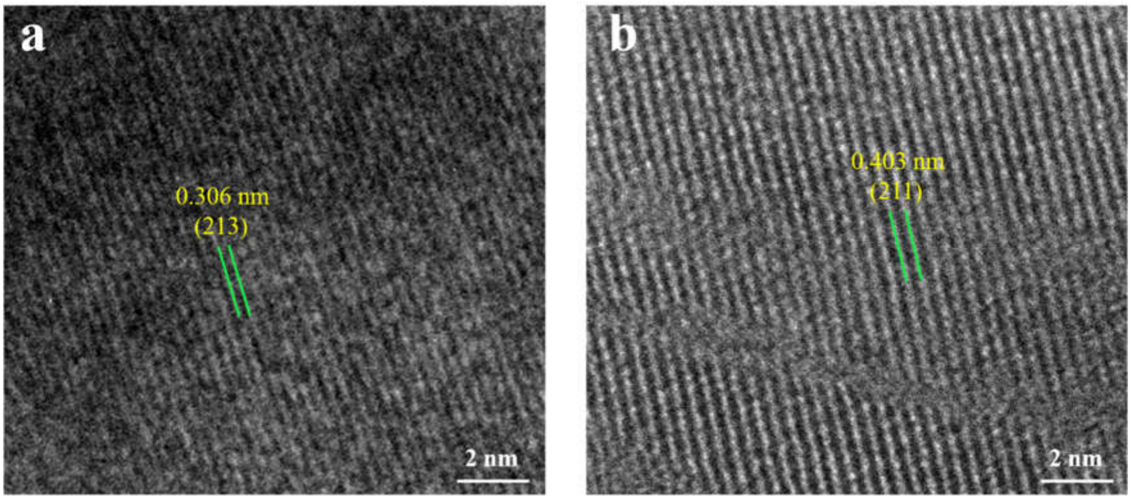

Supplementary Figure 9. Lattice structure after the stability test of $Mn_{7.5}O_{10}Br_3$. HRTEM image of the surface (a) (213) and (b) (211) of the $Mn_{7.5}O_{10}Br_3$ after the stability test.

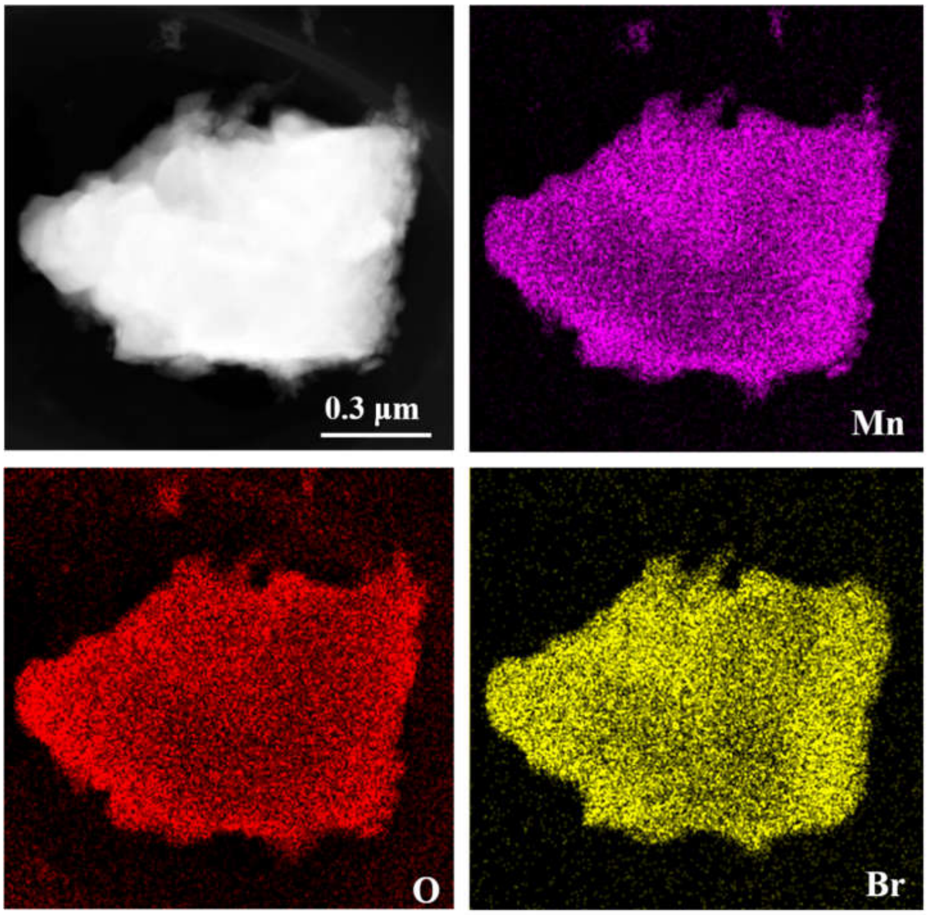

Supplementary Figure 10. Morphology and elemental composition after the stability test. HAADF-STEM image (white) and EDS mapping images (magenta of Mn, red of O, and yellow of Br) of the $Mn_{7.5}O_{10}Br_3$ after the stability test.

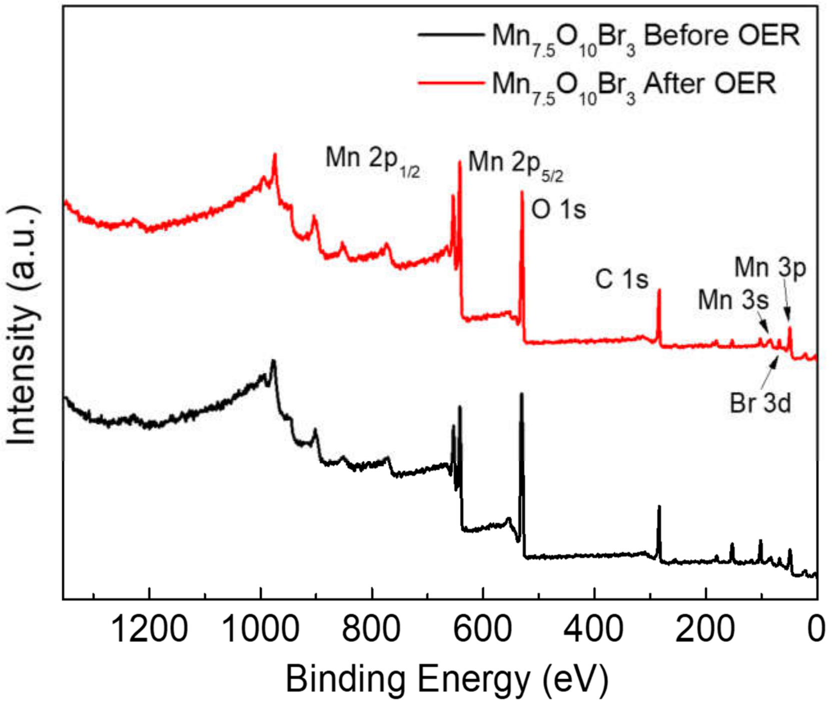

Supplementary Figure 11. Elemental composition after the stability test of $Mn_{7.5}O_{10}Br_3$. Full range XPS spectra of $Mn_{7.5}O_{10}Br_3$ before and after the stability test on carbon cloth.

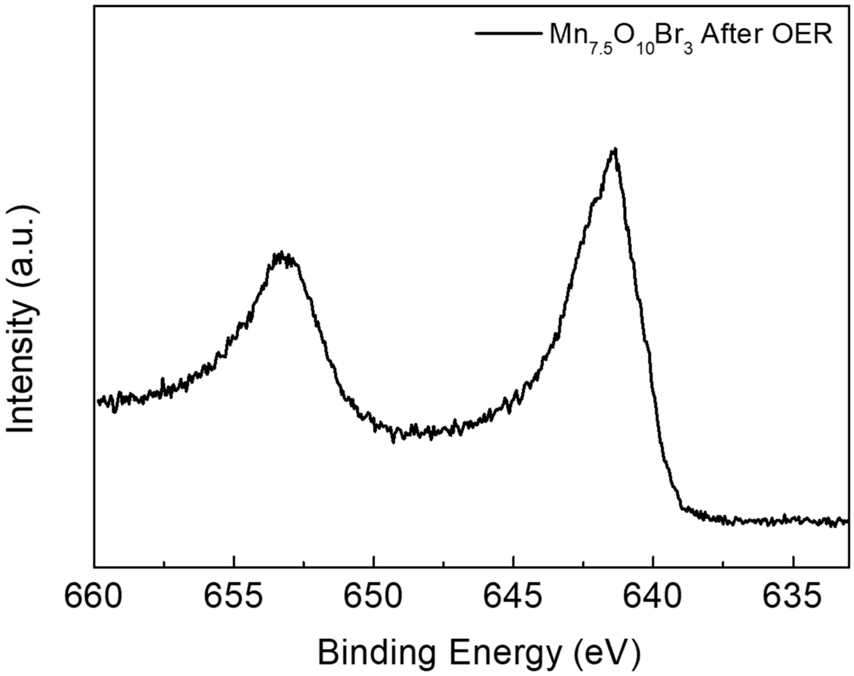

Supplementary Figure 12. Mn element after the stability test of $Mn_{7.5}O_{10}Br_3$. XPS Mn 2p spectra of $Mn_{7.5}O_{10}Br_3$ after the stability test on carbon cloth.

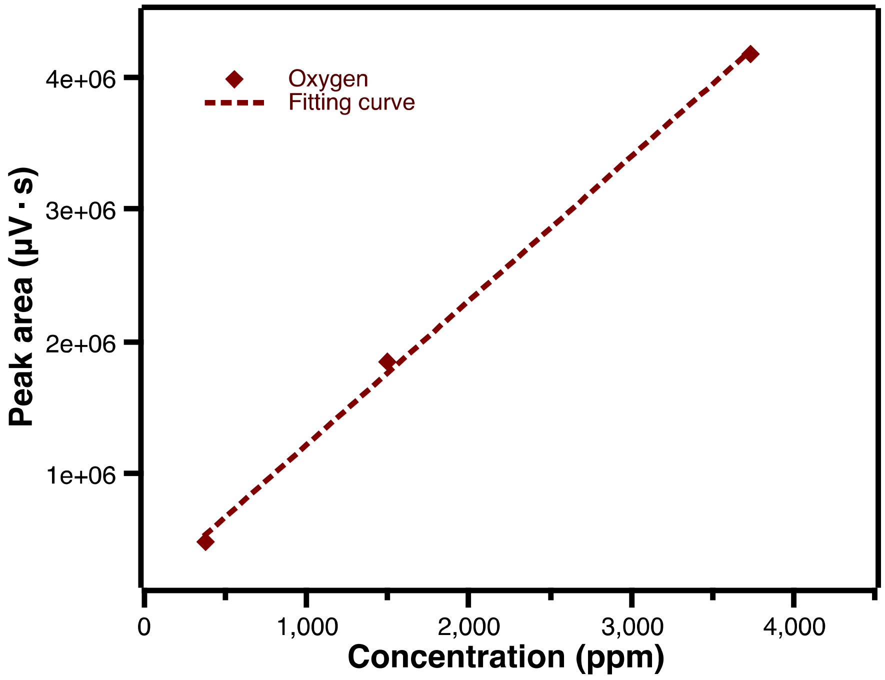

Supplementary Figure 13. Calibration of Faradaic efficiencies test. Calibration curve for oxygen using gas chromatography.

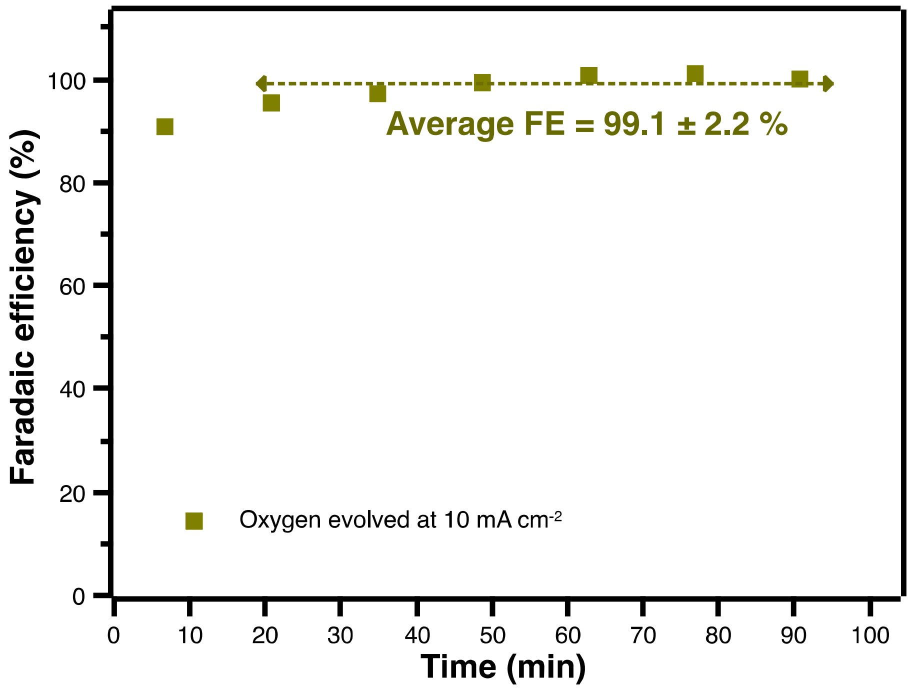

Supplementary Figure 14. Faradaic efficiencies test of $Mn_{7.5}O_{10}Br_3$. Faradaic efficiency of oxygen over 100 min test using $Mn_{7.5}O_{10}Br_3$.

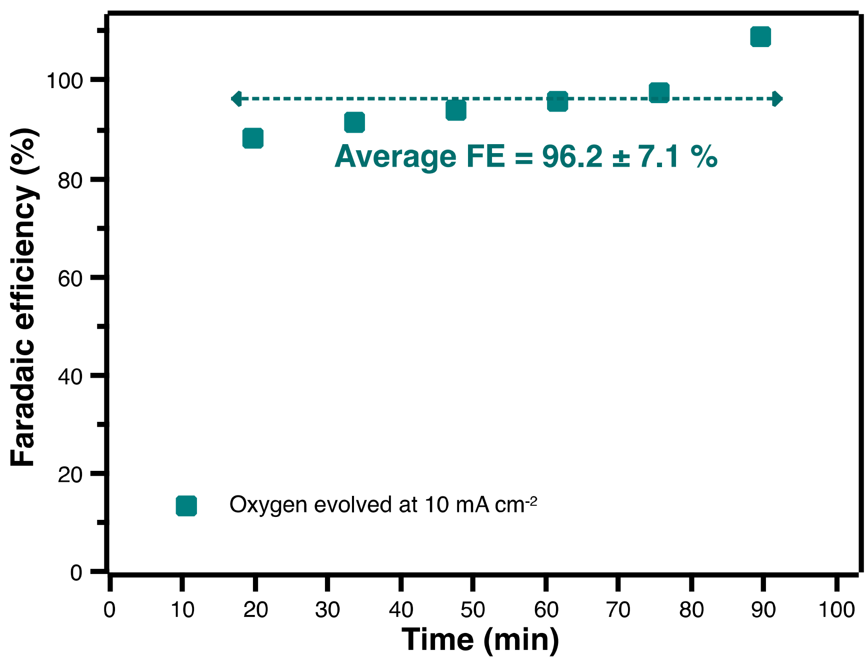

Supplementary Figure 15. Faradaic efficiencies test of $Mn_8O_{10}Cl_3$. Faradaic efficiency of oxygen over 100 min test using $Mn_8O_{10}Cl_3$.

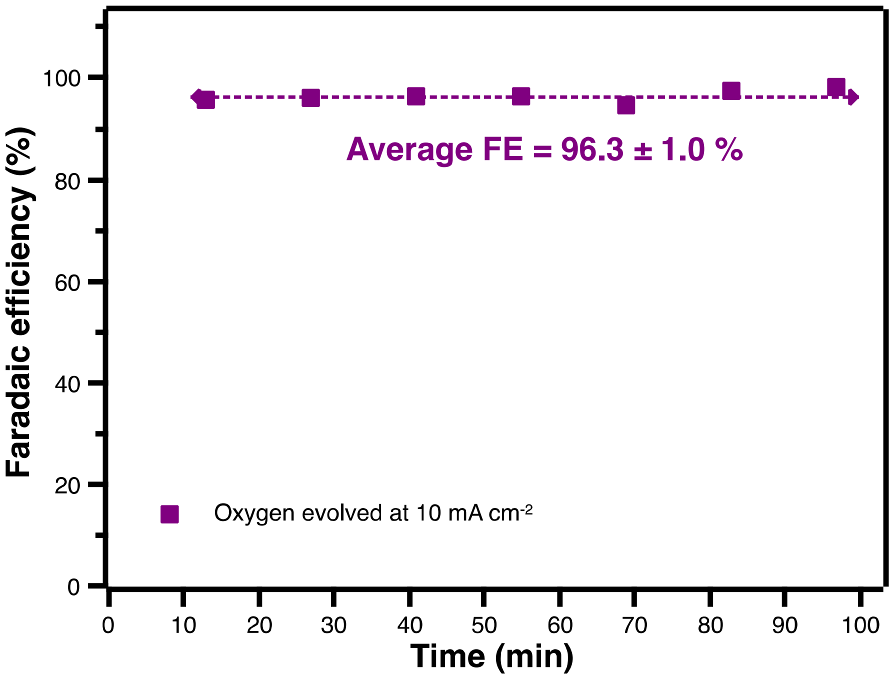

Supplementary Figure 16. Faradaic efficiencies test of γ-MnO_2. Faradaic efficiency of oxygen over 100 min test using γ-MnO_2.

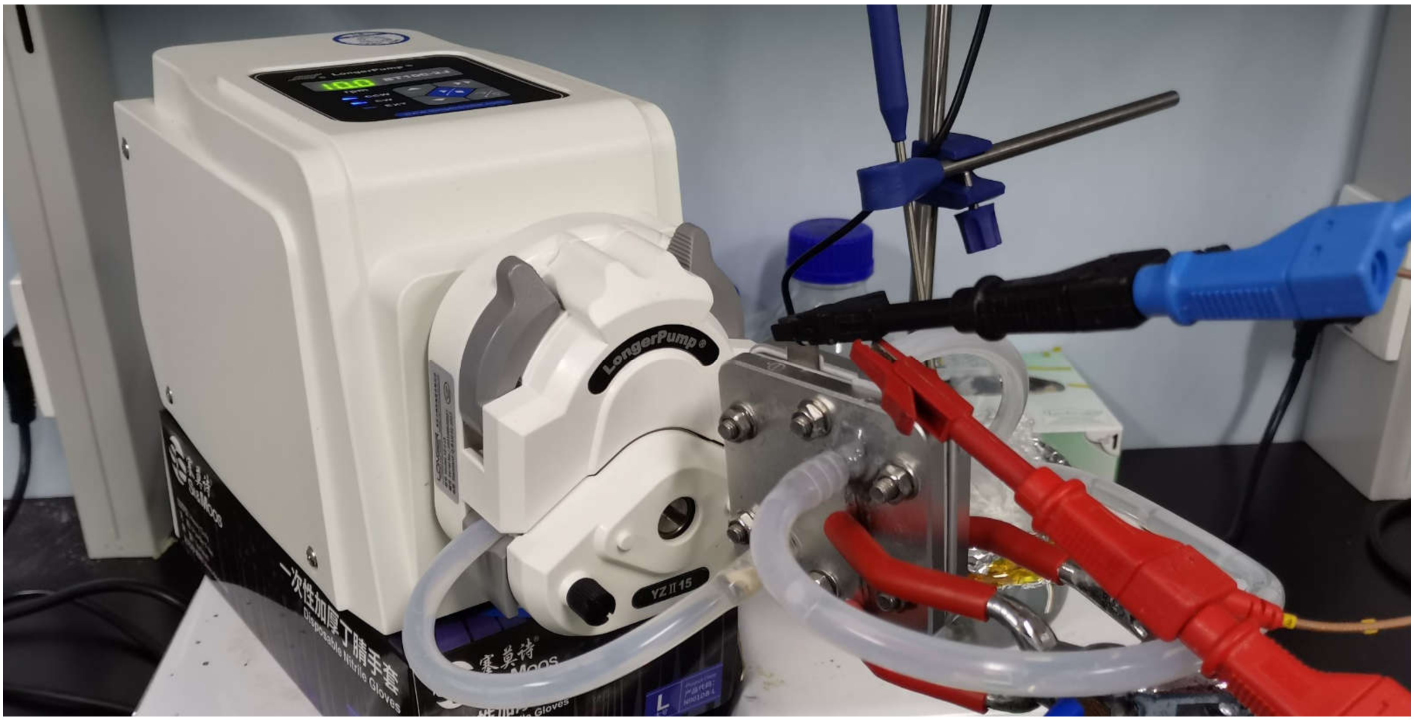

Supplementary Figure 17. PEM test system. Photograph of the PEM set-up used in this work. Keep the temperature of the PEM cell at 50 ^{o}C, and inject the pre-heated DI water into the anode at a flow rate of 10 mL min^{-1}.

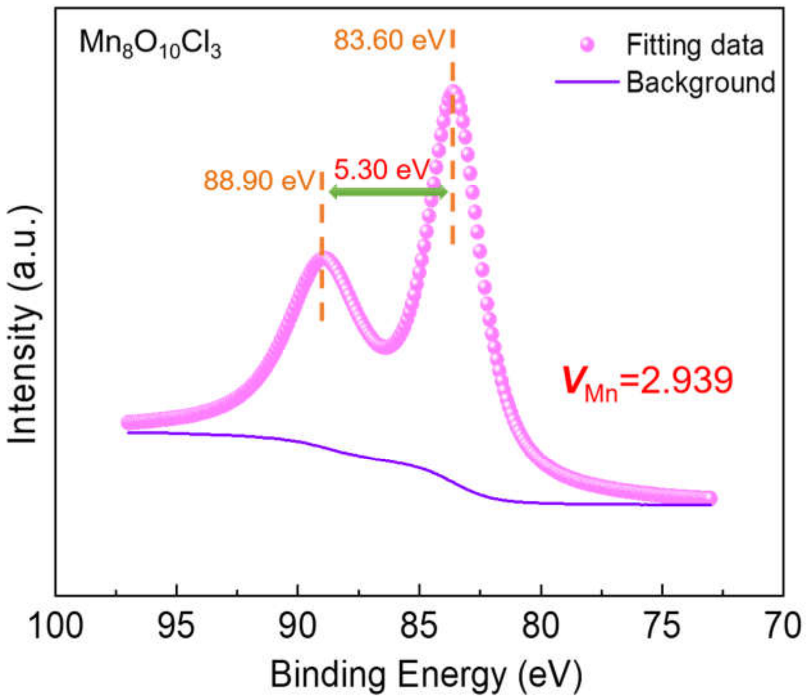

Supplementary Figure 18. Average valence state of Mn element of $Mn_8O_{10}Cl_3$. Mn(3s) XPS spectrum of the $Mn_8O_{10}Cl_3$.

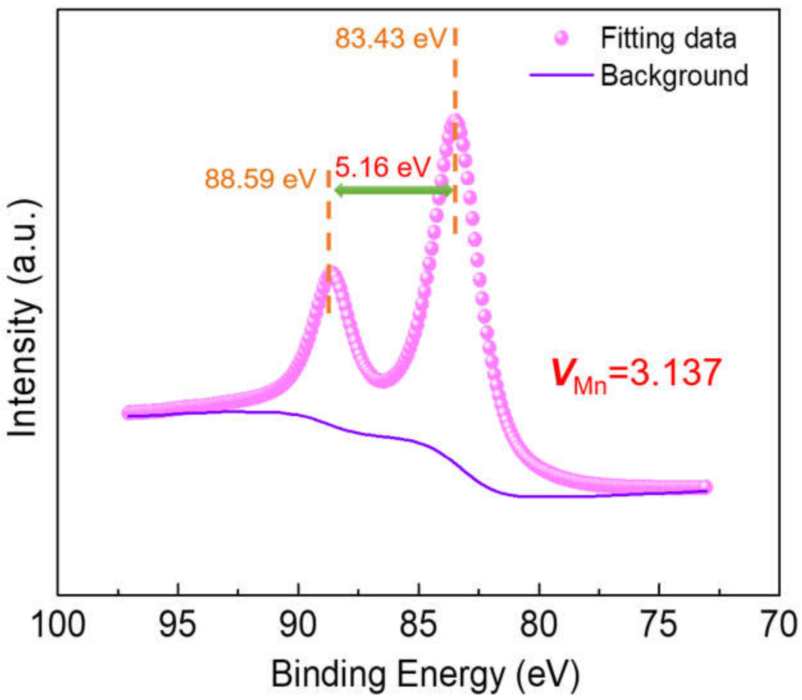

Supplementary Figure 19. Average valence state of Mn element after the stability test of $Mn_{7.5}O_{10}Br_3$. Mn(3s) XPS spectrum of the $Mn_{7.5}O_{10}Br_3$ after the stability test on carbon cloth.

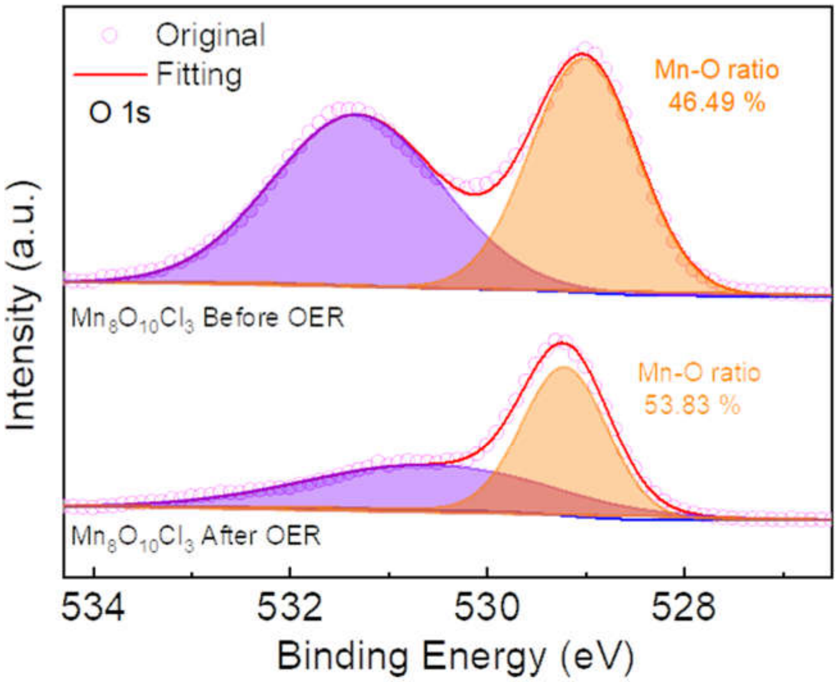

Supplementary Figure 20. XPS of O 1s spectrum before and after OER of $Mn_8O_{10}Cl_3$. XPS O 1s spectra of $Mn_8O_{10}Cl_3$ compared with $Mn_8O_{10}Cl_3$ after OER.

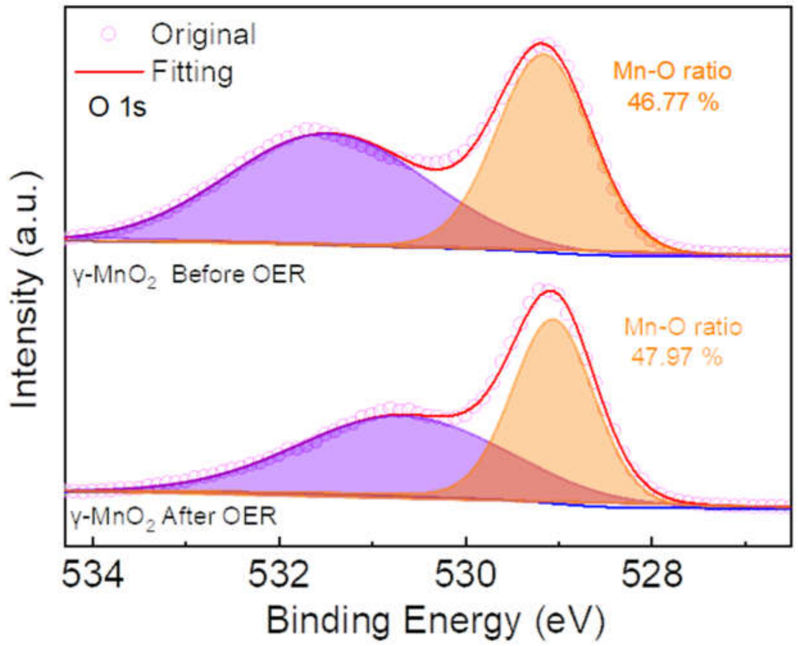

Supplementary Figure 21. XPS of O 1s spectrum before and after OER of γ-MnO_2. High-resolution XPS O 1s spectra of γ-MnO_2 compared with γ-MnO_2 after OER.

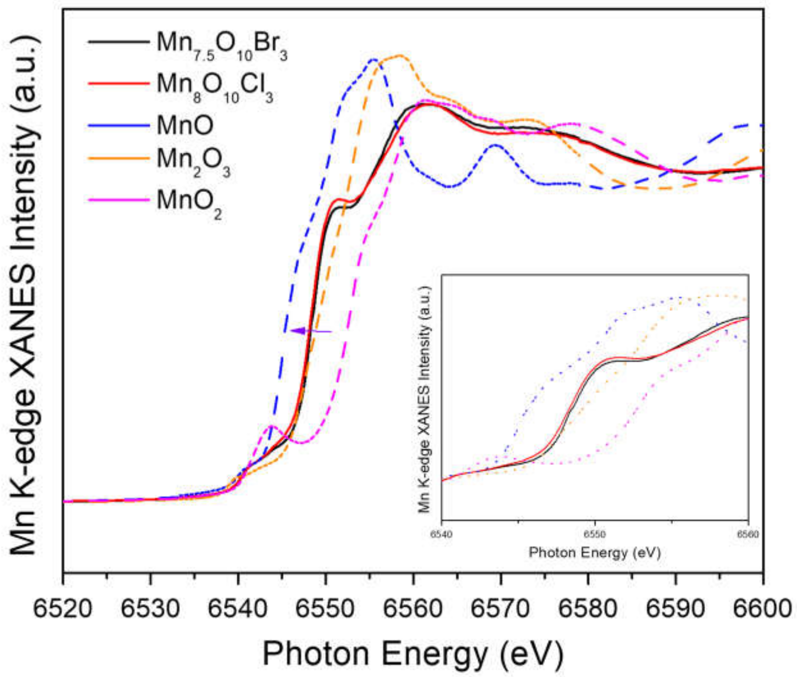

Supplementary Figure 22. XANES spectra of catalysts. Normalized Mn K-edge XANES spectra of $Mn_{7.5}O_{10}Br_3$, $Mn_8O_{10}Cl_3$, and reference materials. The inserted image shows a partial enlargement from 6540 eV to 6550 eV.

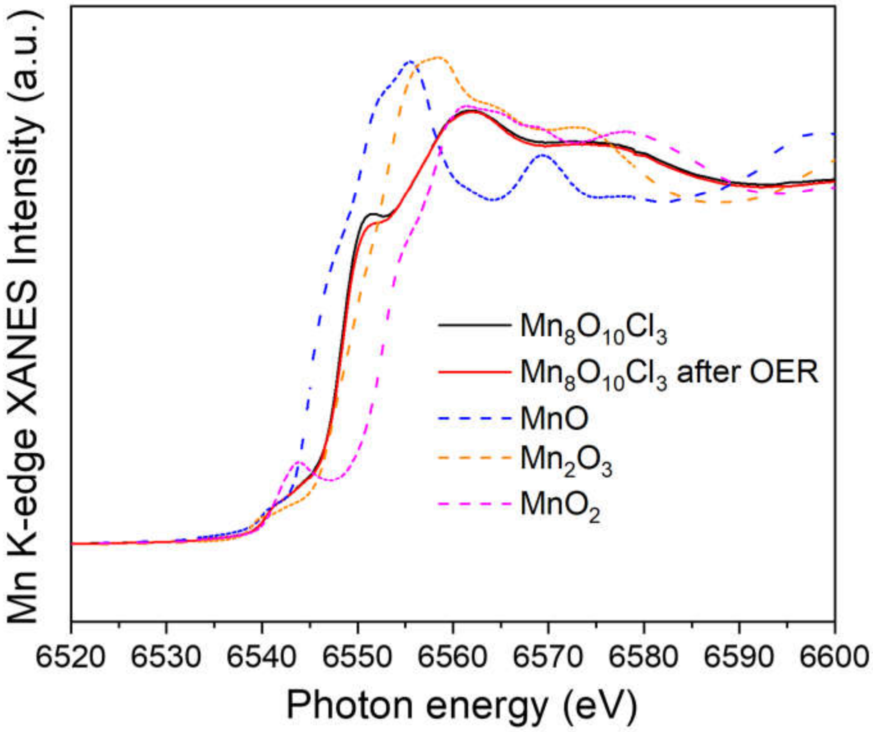

Supplementary Figure 23. XANES spectra of $Mn_8O_{10}Cl_3$ and other catalysts. Normalized Mn K-edge XANES spectra of the $Mn_8O_{10}Cl_3$, $Mn_8O_{10}Cl_3$ after the stability test and reference materials.

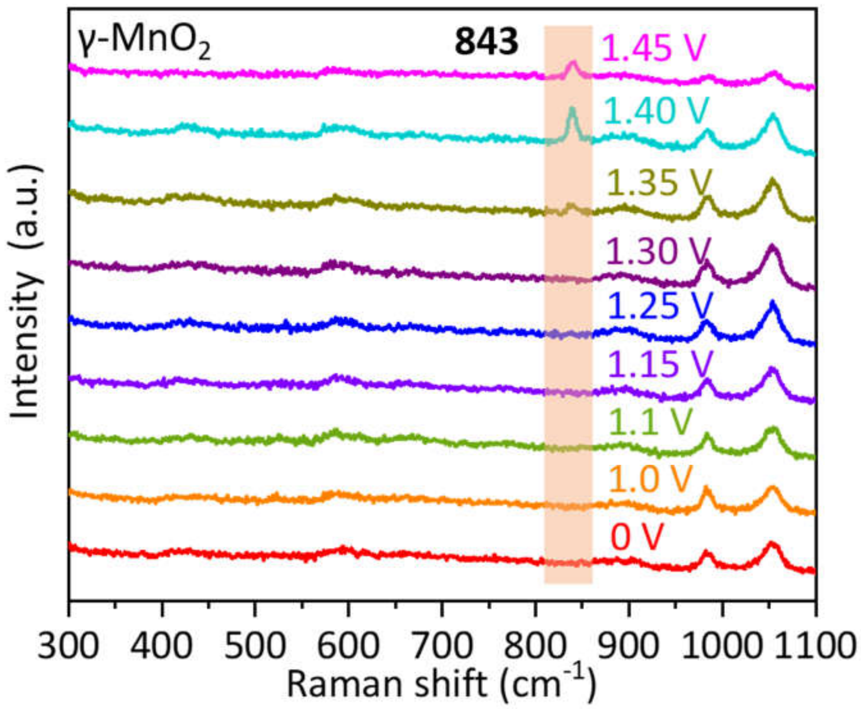

Supplementary Figure 24. Dynamic interface of γ-MnO_2. In-situ Raman spectra of γ-MnO_2 catalyst on a carbon cloth in 0.5 M H_2SO_4 electrolyte under different external applied potential (0-1.45 V).

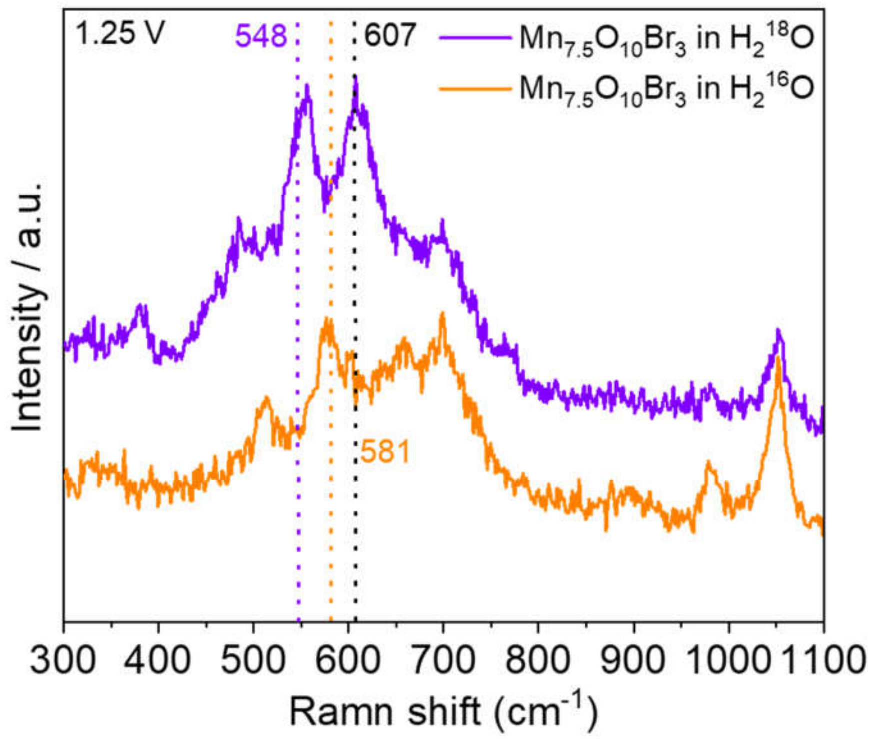

Supplementary Figure 25. Dynamic interface in $H_2{}^{18}O$ and $H_2{}^{16}O$ of $Mn_{7.5}O_{10}Br_3$. In-situ Raman spectra of $Mn_{7.5}O_{10}Br_3$ under external applied potential (1.25 V)

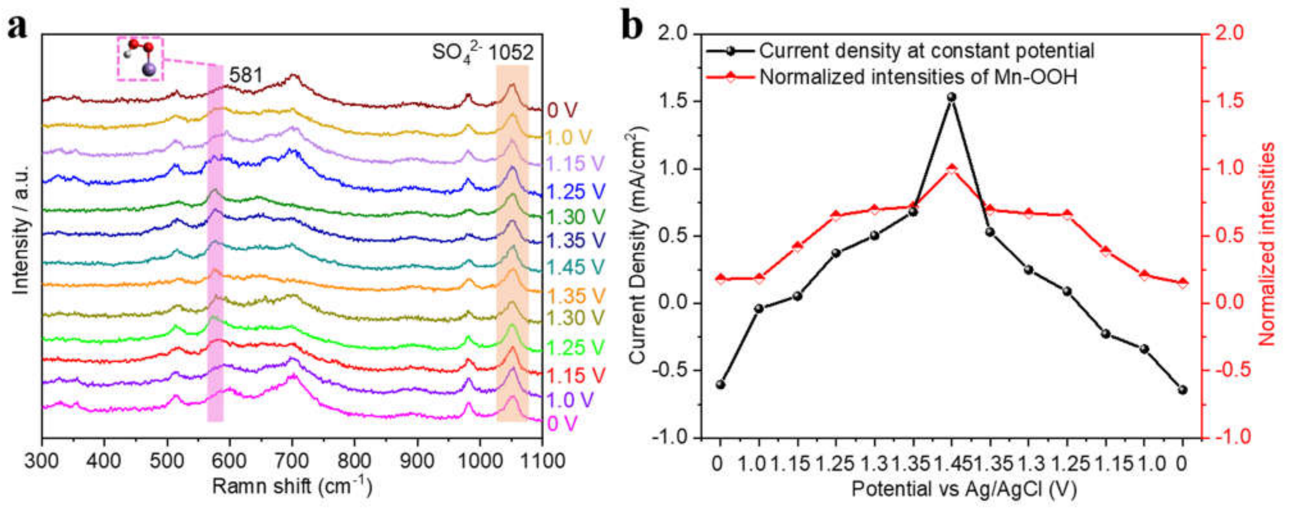

Supplementary Figure 26. By-product exclusion of $Mn_{7.5}O_{10}Br_3$. (a) In-situ normalized Raman spectra (based on the Raman peak area of SO_4^{2-} around 1052 cm^{-1}) of $Mn_{7.5}O_{10}Br_3$ catalyst on a carbon cloth in 0.5 M H_2SO_4 ($H_2{}^{16}O$) electrolyte under a circle of external applied potentials (0V, 1.0V, 1.15V, 1.25V, 1.30V, 1.35V, 1.45V, 1.35V, 1.30V, 1.25V, 1.15V, 1.0V, 0V). (b) The normalized Raman intensity (detail in the methods part) of the stretching mode of Mn-OOH at different potentials (red curve); The current density at constant potential of on a carbon cloth in 0.5 M H_2SO_4 ($H_2{}^{16}O$) electrolyte under different potentials (black curve).

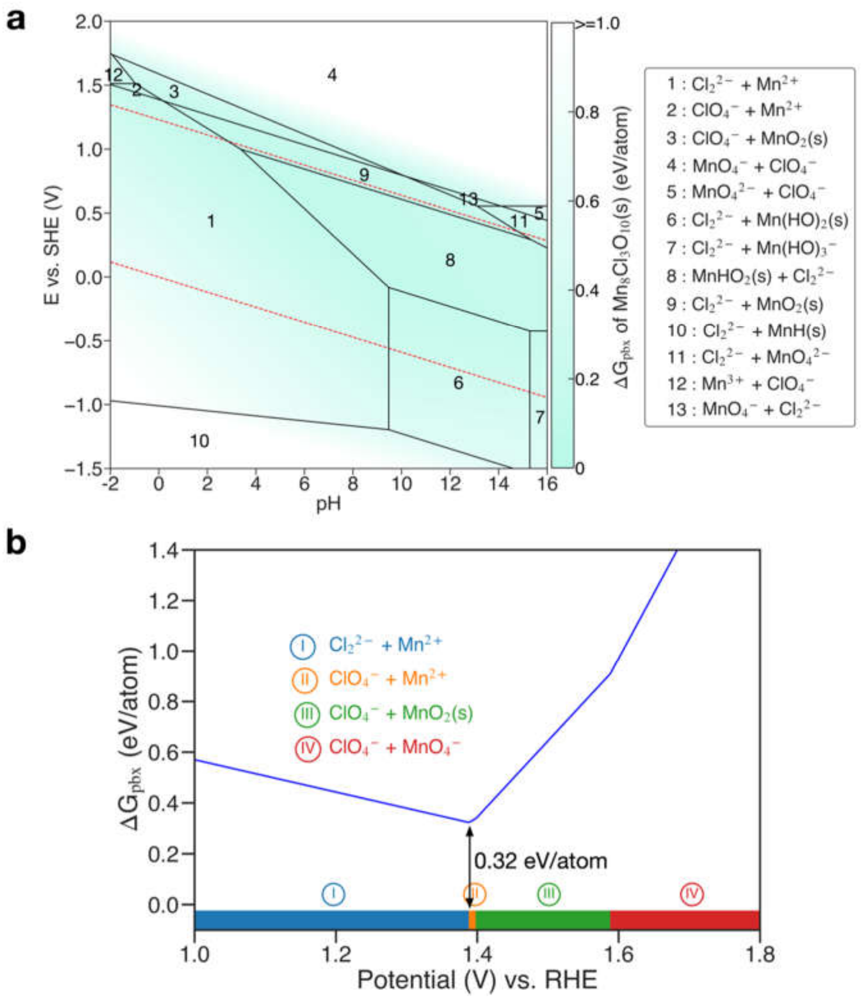

Supplementary Figure 27. Stability of $Mn_8O_{10}Cl_3$. (a) Calculated Mn-O-Cl Pourbaix diagram generated with aqueous ion concentrations 10^{-4} M at 25°C. The Lake blue color gauges the stability of $Mn_8O_{10}Cl_3$ at relevant potential and pH. The water stability window is shown in red dashed line. (b) Calculated Pourbaix decomposition free energy (ΔG_{pbx}) of $Mn_8O_{10}Cl_3$ from the potential 1.0-1.8 V vs. RHE at pH = 0. The projection of ΔG_{pbx} onto the potential axis highlights the stable species at the corresponding regions. Roman numerals are only to index the relevant decomposition products.

Supplementary Discussion

For the activity analysis, we first calculated the surface energies to determine the most energetically favorable surface of the catalysts studied. Based on the calculated surface Pourbaix diagrams, we determined the adsorbate configuration under the OER conditions. The binding energies of adsorbed O and HO were then calculated to evaluate the catalytic activity. The overall procedure is illustrated in Fig. S28. For $Mn_{7.5}O_{10}Br_3$ and $Mn_8O_{10}Cl_3$, (101) surfaces were directly selected based on the HRTEM analysis (Fig. 1). Our calculations indicate that only the O-terminated (101) surfaces of $Mn_{7.5}O_{10}Br_3$ and $Mn_8O_{10}Cl_3$ are stable, with surface energies of 0.06 and 0.05 eV/ Å^2, respectively. In terms of γ-MnO_2, since there is no facet information available from the current experiments or literature, we calculated the surface energies of different possible facets (Supplementary Table 5). We find that the O-terminated (001) surface yields the lowest surface energy of 0.02 eV/ Å^2, compared to the other considered facets. Based on these selected facets, we calculated their surface Pourbaix diagrams by exhaustively screening the possible binding sites for different adsorbates at various coverages. Interestingly, while γ-MnO_2(001) is favorably covered by 0.5 ML H* during OER (Fig. S29a), both $Mn_{7.5}O_{10}Br_3$ and $Mn_8O_{10}Cl_3$ (101) show a notable self-oxidation process at OER conditions (Fig. S29b-c), with the additional O-coverage of 0.50 and 0.64 ML, respectively. This results in a more close-packed oxide surface during OER, with most of the hollow- and bridge-sites filled by additional oxygen with strong O-bonding. Combined with results of bulk Pourbaix analysis, it can be concluded that the formation of MnO_x layer on the surface promotes the long-term stability at operating potentials. On these surfaces identified from surface Pourbaix analysis, all sites were screened for the calculations of O and HO adsorption free energies.

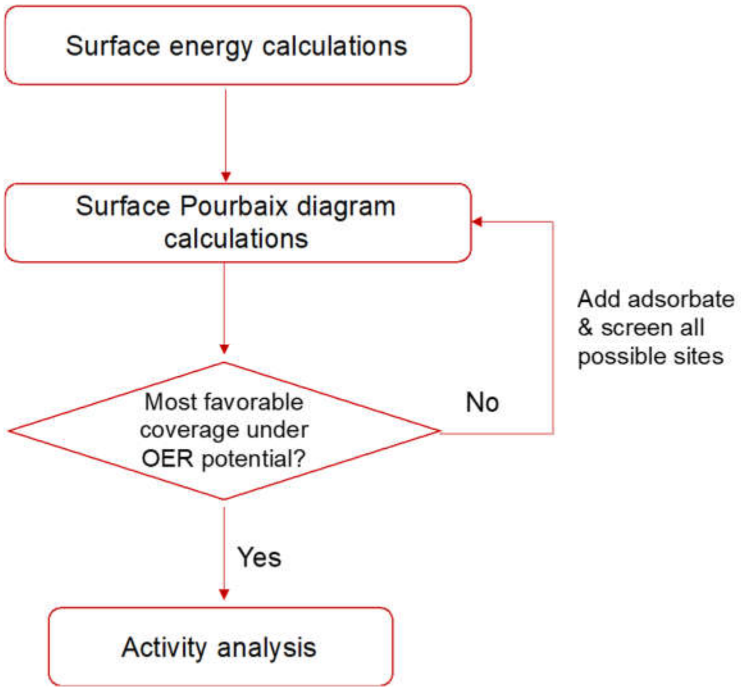

Supplementary Figure 28. Activity analysis. Flow chart of the OER activity analysis in this paper consists of surface energy calculations, surface Pourbaix diagram calculations, and activity analysis based on the most favorable coverage indicated by the surface Pourbaix diagram analysis. This flow chart describes the analytical procedure of the theoretical sections in this paper, including the analysis of surface stability, surface state, and activity analysis based on the most favorable surface state at electrochemical conditions.

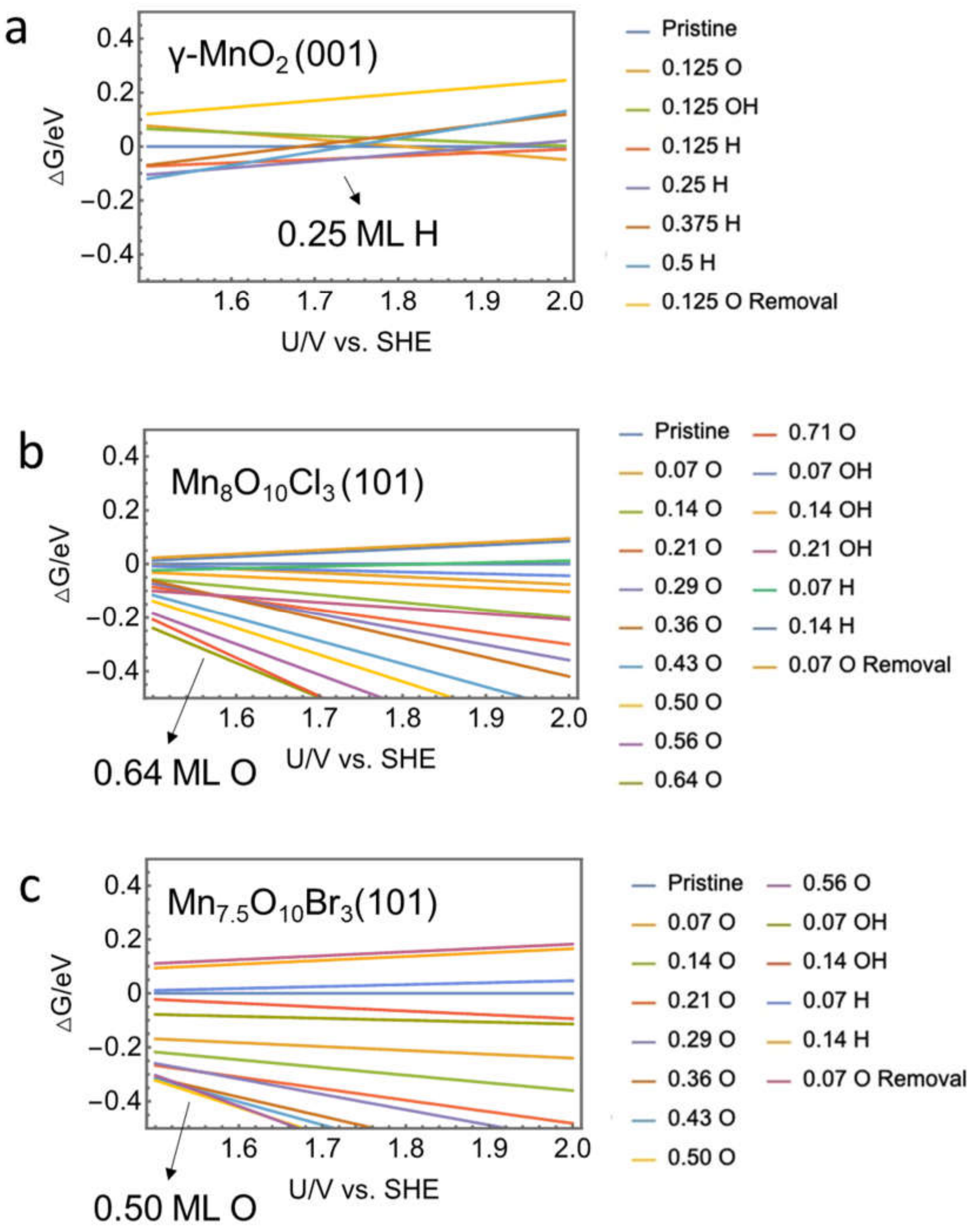

Supplementary Figure 29. Activity analysis. Surface Pourbaix diagram calculations on (a) γ-MnO_2(001), (b) $Mn_8O_{10}Cl_3$(101), and (c) $Mn_{7.5}O_{10}Br_3$(101), with a pH of 0.29.

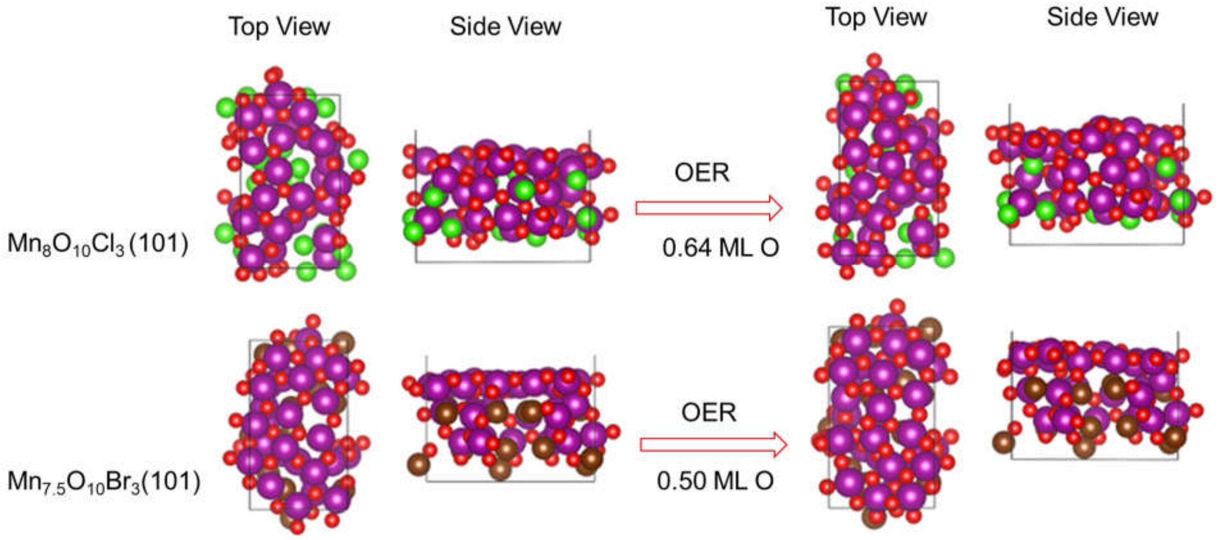

Supplementary Figure 30. Activity analysis. Self-oxidation on $Mn_8O_{10}Cl_3$(101) and $Mn_{7.5}O_{10}Br_3$(101) during OER as indicated by the surface Pourbaix diagram analysis. Purple, red, green, and brown spheres represent Mn, O, Cl, and Br, respectively.

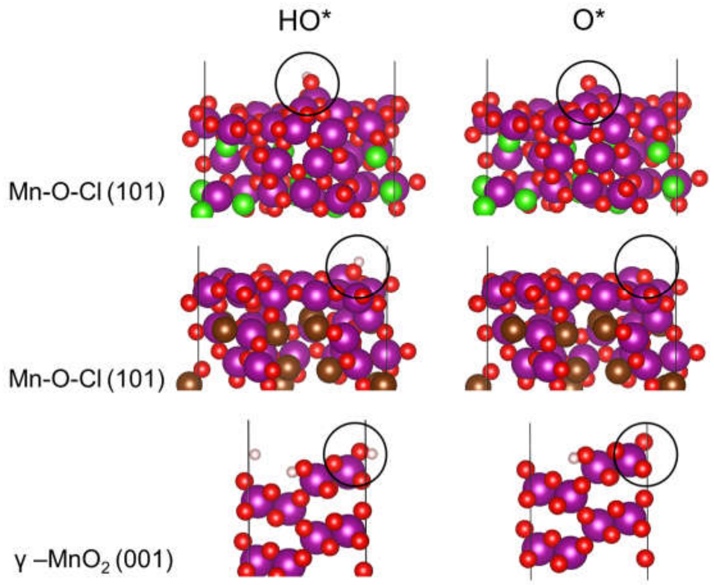

Supplementary Figure 31. Activity analysis. The most favorable binding configuration of key OER adsorbate on the surfaces with the states indicated by the surface Pourbaix diagram analysis. Purple, red, green, brown, and pink spheres represent Mn, O, Cl, Br, and H, respectively.

Supplementary Table 1. Summary of noble-metal-free OER catalyst performance. The data of Ir-based noble metal-based catalysts were also collected for reference and shown in *italics*.

Catalyst (Electrolyte)	Overpotential (mV at 10 mA cm^{-2})	Substrate	Stability (at 10 mA cm^{-2})	Reference
**γ-MnO_2 (H_2SO_4 0.5M)**	**413 ± 5**	**CC**	-	**This Work**
**$Mn_8O_{10}Cl_3$ (H_2SO_4 0.5M)**	**368 ± 5**	**CC**	**70 h**	**This Work**
**$Mn_{7.5}O_{10}Br_3$ (H_2SO_4 0.5M)**	**295 ± 5**	**CC**	**500 h on FTO<br>300 h @ 100 mA cm^{-2} in PEM**	**This Work**
Ba[Co-POM] (H_2SO_4 1M)	500	CP	24 h @ 250 mV	(2)
$Ni_{0.5}Mn_{0.5}Sb_{1.7}O_y$ (H_2SO_4 1M)	672 ± 9	ATO	168 h	(3)
Mn-rich rutile $Mn_xSb_{1-x}O_z$ (H_2SO_4 1M)	580	Pt/Ti/SiO2/Si wafer	~30 h	(4)
Co_3O_4 (H_2SO_4 0.5M)	570	-	10 h	(5)
$CoFePbO_x$ (H_2SO_4 0.05M)	700	FTO	10 h	(6)
Ag-doped Co_3O_4 (H_2SO_4 0.5M)	470	FTO	10 h	(7)
C-coated Co_3O_4 (H_2SO_4 0.5M)	370	CP	86.8 @ 100 mA cm^{-2}	(8)
DN gels (InFeCo-CCP) (H_2SO_4 0.5M)	450 @ 1mA cm^{-2}	GCE	3000 cycles and 40 h @ 5 mA cm^{-2}	(9)
γ-MnO_2 (H_2SO_4 1M)	428 ± 5	CP	8000 h on FTO<br>12 h @ 100 mA cm^{-2} in PEM	(10)
$IrO_x/SrIrO_3$ ($HClO_4$ 0.1M)	*270-290*	*CW*	*30 h*	(11)
IrO_2 ($HClO_4$ 0.1M)	*380*	*GCE*	-	(12)

CC: carbon cloth electrode, CP: carbon paper electrode, GCE: glassy carbon electrode, CW: Copper wire

Notes: $NiMnSbO_y$ is stable over 168 hrs, but its overpotential is very large, i.e. more than 2.2 times that of $Mn_{7.5}O_{10}Br_3$. The overpotential of γ-MnO_2 increases *ca.* 80 mV with 8000 hrs operation at 10 mA/cm^2. $Mn_{7.5}O_{10}Br_3$ exhibits a 44 mV overpotential increase with 500 hrs operation at 10 mA/cm^2. The stability test of the $Mn_{7.5}O_{10}Br_3$ catalyst on FTO is still ongoing. In PEM cell stability tests, the γ-MnO_2 catalyst become inactivated after 12 hrs with a current density of 100 mA/cm^2 at 25 ° C. In stark contrast, $Mn_{7.5}O_{10}Br_3$ exhibits no stability decrease over 12 hrs operation at 100 mA/cm^2 and 50°C in PEM cell. The ongoing PEM cell testing show that the voltage of $Mn_{7.5}O_{10}Br_3$ only increases 124 mV after 300 hrs operation.

Supplementary Table 2. ICP measurement of Mn ions in the electrolyte before and after stability test.

Sample (0.5 M H_2SO_4 Electrolyte)	Dissolved manganese
Before stability test	11.88 ppb[c]
$Mn_{7.5}O_{10}Br_3$ (after stability test)	1.05 ppm[b]
$Mn_{7.5}O_{10}Br_3$ counter electrode[a] (after stability test)	12.82 ppb[c]
$Mn_8O_{10}Cl_3$ (after stability test)	8.74 ppm[b]
$Mn_8O_{10}Cl_3$ counter electrode[a] (after stability test)	13.28 ppb[c]
γ-MnO_2 (after stability test)	6.86 ppm[b]
γ-MnO_2 counter electrode[a] (after stability test)	15.20 ppb[c]

a: obtained by soaking the counter electrode
b: detected by ICP-OES
c: detected by ICP-MS

Supplementary Table 3. Detailed XPS O 1s data of catalysts before and after OER test.

Sample	Mn-O		Total	Mn-O ratio (%)
$Mn_{7.5}O_{10}Br_3$ before OER	12740.67	22707.21	35447.88	35.94
$Mn_{7.5}O_{10}Br_3$ after OER	14528.76	14485.44	29014.2	50.07
$Mn_8O_{10}Cl_3$ before OER	20802.25	23946.44	44748.69	46.49
$Mn_8O_{10}Cl_3$ after OER	10520.2	9021.633	19541.83	53.83
γ-MnO_2 before OER	20806.32	23680.54	44486.86	46.77
γ-MnO_2 after OER	16840.49	18265.25	35105.74	47.97

Supplementary Table 4. Comparison of the OER potentials between experiments and theoretical models at 10 mA/cm^2.

Potential @ 10 mA/cm^2	Mn-O-Br	Mn-O-Cl	γ-MnO_2
Experiment (V)	1.52	1.60	1.65
Theory (V)	1.74	1.80	1.93
Deviation (V)	-0.22	-0.20	0.32

Supplementary Table 5. Calculated surface energies for γ-MnO_2. Only the stable surfaces after structural relaxation are shown.

γ-MnO_2	(001)-O	(010)-O	(100)-Mn	(100)-O	(110)-O
Surface energy (eV/ $Å^2$)	0.02	0.08	0.15	0.15	0.08

References

1. D. D. Wagman et al. NBS Technical note 270, Washington; 1968-1971
2. Blasco-Ahicart, M. et al. Polyoxometalate electrocatalysts based on earthabundant metals for efficient water oxidation in acidic media. *Nat. Chem.* **10**, 24-30 (2018).
3. Moreno-Hernandez, I. A. Crystalline Nickel Manganese Antimonate as a Stable Water Oxidation Catalyst in Aqueous 1.0 M H_2SO4. et al. *Energ. Environ. Sci.* **10**, 2103-2108 (2017).
4. Zhou, L. et al. Rutile Alloys in the Mn−Sb−O System Stabilize Mn^{3+} To Enable Oxygen Evolution in Strong Acid. *ACS Catal.* **8**, 10938-10948 (2018).
5. Mondschein, J. S. et al. Crystalline Cobalt Oxide Films for Sustained Electrocatalytic Oxygen Evolution under Strongly Acidic Conditions. *Chem. Mater.* **29**, 950-957 (2017).
6. Chatti, M. et al. Intrinsically stable in situ generated electrocatalyst for long-term oxidation of acidic water at up to 80 °C. *Nat. Catal.* **2**, 457-465 (2019).
7. Yan, K.-L. et al. Probing the active sites of Co_3O_4 for the acidic oxygen evolution reaction by modulating the Co^{2+}/Co^{3+} ratio. *J. Mater. Chem. A* **6**, 5678-5686 (2018).
8. Yang, X. et al. Highly acid-durable carbon coated Co_3O_4 nanoarrays as efficient oxygen evolution electrocatalysts. *Nano Energy* **25**, 42-50 (2016).
9. Fang, Z. et al. Hybrid Organic−Inorganic Gel Electrocatalyst for Stable Acidic Water Oxidation. *ACS nano*. **13**, 14368-14376 (2019).
10. Li, A. *et al.* Stable Potential Windows for Long-Term Electrocatalysis by Manganese Oxides Under Acidic Conditions. *Angew. Chem. Int. Ed.* **58**, 5054-5058 (2019).
11. Seitz, L. C. et al. A highly active and stable $IrO_x/SrIrO_3$ catalyst for the oxygen evolution reaction. *Science* **35**, 1011-1014 (2016).
12. Yao, Y. et al. Engineering the electronic structure of single atom Ru sites via compressive strain boosts acidic water oxidation electrocatalysis. *Nat. Catal.* **2**, 304-313 (2019).
