# Supplementary material for: Efficient and stable noble-metal-free catalyst for acidic water oxidation
Source: Nat Commun. 2022 Apr 28;13:2294. doi: 10.1038/s41467-022-30064-6 (PMC9050677; doi:10.1038/s41467-022-30064-6)
Supplement: Supplementary file 2 — Description of Additional Supplementary Files [file 41467_2022_30064_MOESM2_ESM.pdf]

File name: Supplementary Movie 1

Description: The stability of $Mn_{7.5}O_{10}Br_3$ was recorded for 388 hrs under the current density of $10mA/cm^2$ in 0.5 M H_2SO_4 solution.
